# Selective Gold Precipitation by a Tertiary Diamide Driven by Thermodynamic Control

**DOI:** 10.1021/acs.inorgchem.4c01279

**Published:** 2024-05-09

**Authors:** Susanna S. M. Vance, Mateusz Mojsak, Luke M. M. Kinsman, Rebecca Rae, Caroline Kirk, Jason B. Love, Carole A. Morrison

**Affiliations:** EaStCHEM School of Chemistry, University of Edinburgh, Edinburgh EH9 3FJ, United Kingdom

## Abstract

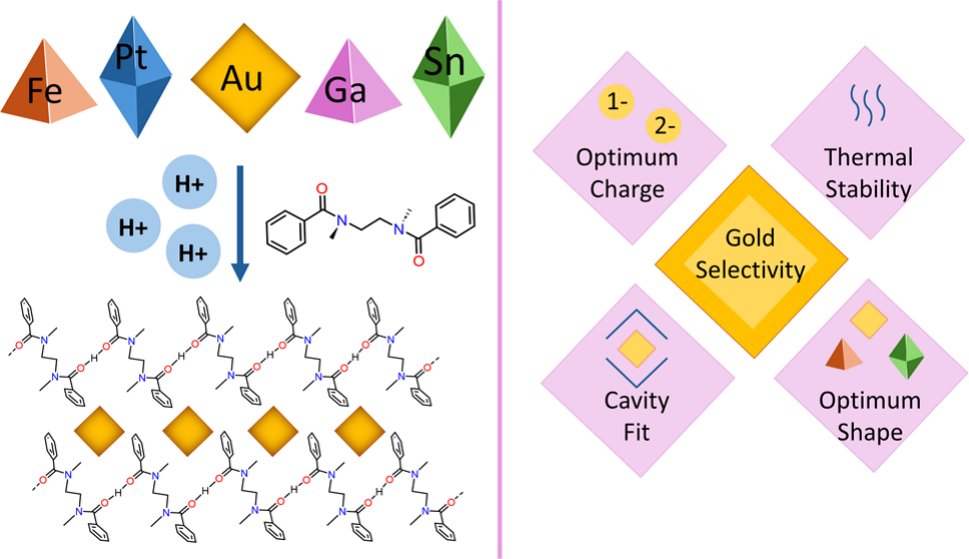

The simple diamide ligand **L** was previously shown to selectively precipitate gold from acidic solutions typical of e-waste leach streams, with precipitation of gallium, iron, tin, and platinum possible under more forcing conditions. Herein, we report direct competition experiments to afford the order of selectivity. Thermal analysis indicates that the gold-, gallium-, and iron-containing precipitates present as the most thermodynamically stable structures at room temperature, while the tin-containing structure does not. Computational modeling established that the precipitation process is thermodynamically driven, with ion exchange calculations matching the observed experimental selectivity ordering. Calculations also show that the stretched ligand conformation seen in the X-ray crystal structure of the gold-containing precipitate is more strained than in the structures of the other metal precipitates, indicating that intermolecular interactions likely dictate the selectivity ordering. This was confirmed through a combination of Hirshfeld, noncovalent interaction (NCI), and quantum theory of atoms in molecules (QTAIM) analyses, which highlight favorable halogen···halogen contacts between metalates and pseudo-anagostic C–H···metal interactions in the crystal structure of the gold-containing precipitate.

## Abstract 2

Selective recovery of gold from e-waste leach streams rationalized by a combined experimental and computational approach; competition experiments afford the order of selectivity, while solid-state DFT calculations provide relative thermodynamic stabilities. Hirshfeld surfaces, noncovalent interaction (NCI) plots and quantum theory of atoms in molecules (QTAIM) rationalize the superior encapsulation of the gold metalate.

## Introduction

Metals are a key resource in today’s society,^1,2^ but constant innovation and consumerism confine them to a growing stockpile of obsolete electrical and electronic equipment.^3,4^ Referred to as e-waste, it is now the fastest-growing hazardous solid-waste stream in the world, with an estimated value of $57 billion USD.^5−7^ As the waste stream grows, natural sources of many key metals are diminishing, rendering the current process unsustainable.^8,9^ Best estimates suggest that only *ca*. 17% of e-waste is recycled through environmentally sound practices, with the remainder either directed to landfill or shipped to developing countries for rudimentary processing,^7,10−12^ in the latter case, the crude practices adopted result in the release of toxic byproducts which threaten human health and the environment.^13−15^ Thus, the sustainable management and recycling of e-waste is crucial in tackling a global shortage of key metals, minimizing environmental damage, and realizing a circular economy.^16−20^

The complex nature of e-waste mandates that any viable recycling process must be highly selective for the target metal.^21^ A variety of extractive metallurgical techniques have been adapted from the processing of natural mineral ores to e-waste to this end.^22^ Hydrometallurgical processes, which involve the dissolution and subsequent separation of metals, offer significant advantages over pyrometallurgical processes, which release substantial amounts of toxic byproducts.^22^ Hydrometallurgical separation processes that utilize coordination and supramolecular chemistry facilitate careful tuning of ligand design, affording selectivity based on size effects or molecular recognition.^23,24^ Metal speciation is therefore critical in the design of novel ligands, which can vary depending on the leaching conditions. For chloride leaching, which is widely employed in the recovery of base and precious metals, the metals present as chloridometalates, which vary in their size, shape, and charge. These differences can be exploited to separate and purify metals using processes that are more environmentally benign than the long-standing methods applied in gold ore refining that rely on cyanide leaching or mercury alloying.^25−27^

Solvent extraction, which exploits coordination and supramolecular chemistry principles, has found widespread use in the recovery of metals from primary and secondary sources.^28^ In the field of gold recovery, amides have found application as selective gold extractants,^29−31^ with selectivity following the Hofmeister bias, meaning that metalates of lower charge are more readily extracted due to their smaller energies of hydration.^32^ Selectivity can also be achieved with adsorbents and precipitants, such as porous porphyrin polymers that utilize size discrimination,^33,34^ or metal–organic frameworks (MOFs)^35,36^ and covalent-organic frameworks (COFs)^37^ that afford separation through selective reduction of gold. Shape can also play an important role in achieving metalate selectivity, with cucurbit[*n*]urils and cyclodextrins functioning as selective gold precipitants based on molecular recognition.^38−43^ Acyclic amides have also been employed to the same effect, with the shape of the tetrachloroaurate anion maximizing host–guest interactions.^44,45^

**Figure 1:**
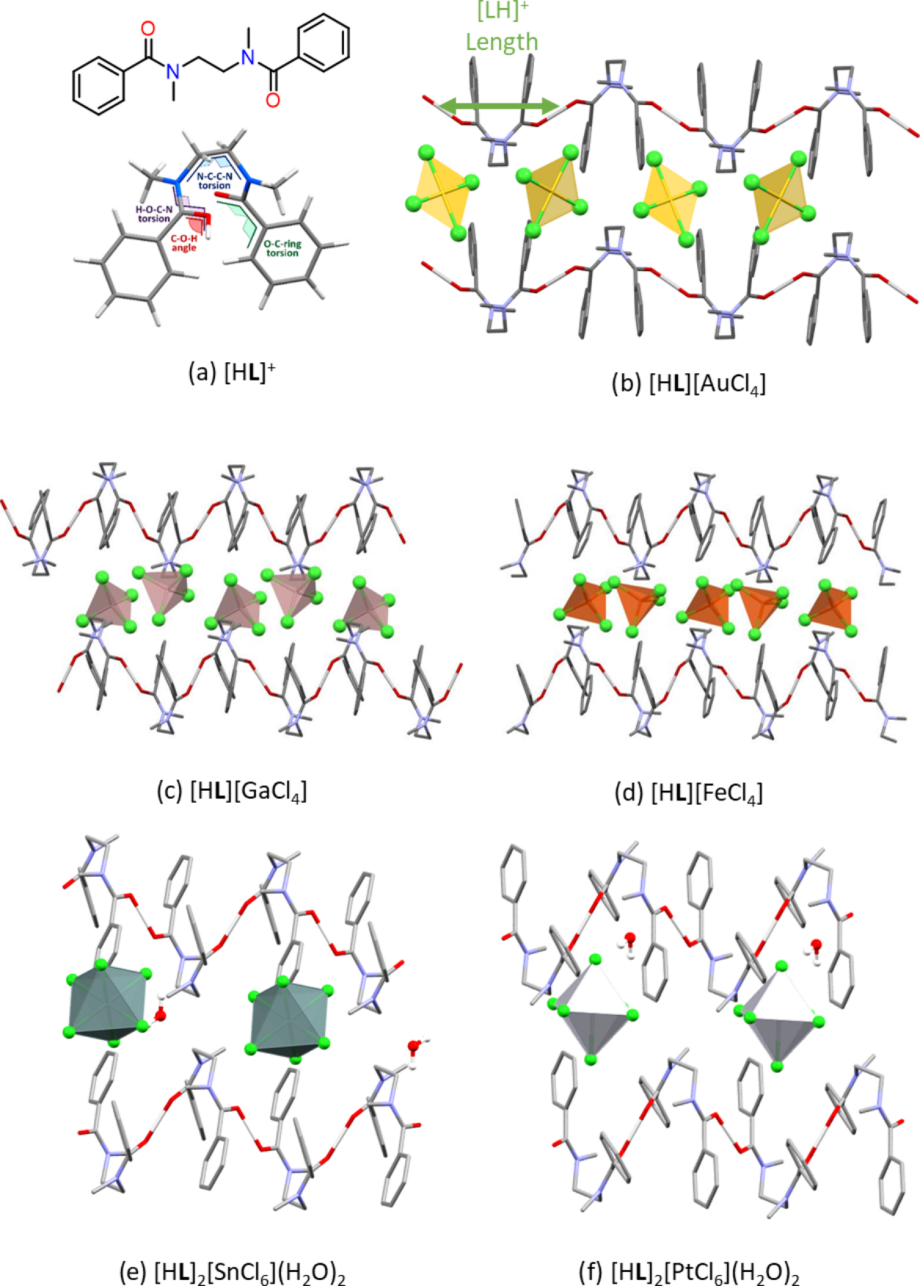
(a) Top = chemical structure of **L**, bottom = single unit of [H**L**]^+^ coil extracted from the X-ray crystal structure of the gold complex, depicting the bond angles and torsions presented in Table 1. (b–f) Geometries of the precipitated species obtained from single-crystal X-ray diffraction studies.^46^ Atom colors: gray = carbon, white = hydrogen, blue = nitrogen, red = oxygen, green = chlorine, yellow = gold, pink = gallium, orange = iron, dark green = tin, silver = platinum.

Metal-selective precipitants that operate directly on the leach solution are of particular interest as their use negates the need for organic solvents that are necessary in solvent extraction processes, making the overall recovery process more environmentally benign. We have previously reported one such process, whereby a simple tertiary diamide ligand (**L**, Figure 1a) afforded high selectivity for gold from mixed-metal acidic solutions through formation of intermolecular proton chelated coils (Figure 1b–f).^46^ The reasons for the selectivity shown by **L** remained elusive, however, which obscured the prospects of developing rational design criteria for similar processes. We address this here through a series of competition experiments to establish the exact order of metal selectivity exhibited by **L**. This is then followed by an experimental thermodynamic study using differential scanning calorimetry (DSC) coupled to powder X-ray diffraction (PXRD) to give insight into the thermal stability of the series of metal-containing precipitates. Finally, we report an in-depth computational modeling study that explores the reasons why [H**L**][AuCl_4_] forms preferentially over [H**L**][GaCl_4_], [H**L**][FeCl_4_], [H**L**]_2_[SnCl_6_](H_2_O)_2_ and [H**L**]_2_[PtCl_6_](H_2_O)_2_.

## Results and Discussion

### Determination of Selectivity Order

Previous work has shown that, upon protonation, **L** preferentially precipitates [AuCl_4_]^−^ over other metalates from 2 M HCl solution. Under more forcing conditions (6 M HCl and a 10-fold excess of **L**), precipitation of [FeCl_4_]^−^, [GaCl_4_]^−^, [SnCl_6_]^2–^, and [PtCl_6_]^2–^ was also observed, though selectivity for gold at 6 M HCl can be retained if a stoichiometric equivalent of **L** is used instead of an excess.^46^ Attempted precipitation of [PdCl_4_]^2–^ which presents as a square-planar metalate in common with [AuCl_4_]^−^, from a mixed-metal feedstock gave a negative result, which suggests that charge and metalate geometry are important factors that govern the precipitation process.^46^

**Figure 2:**
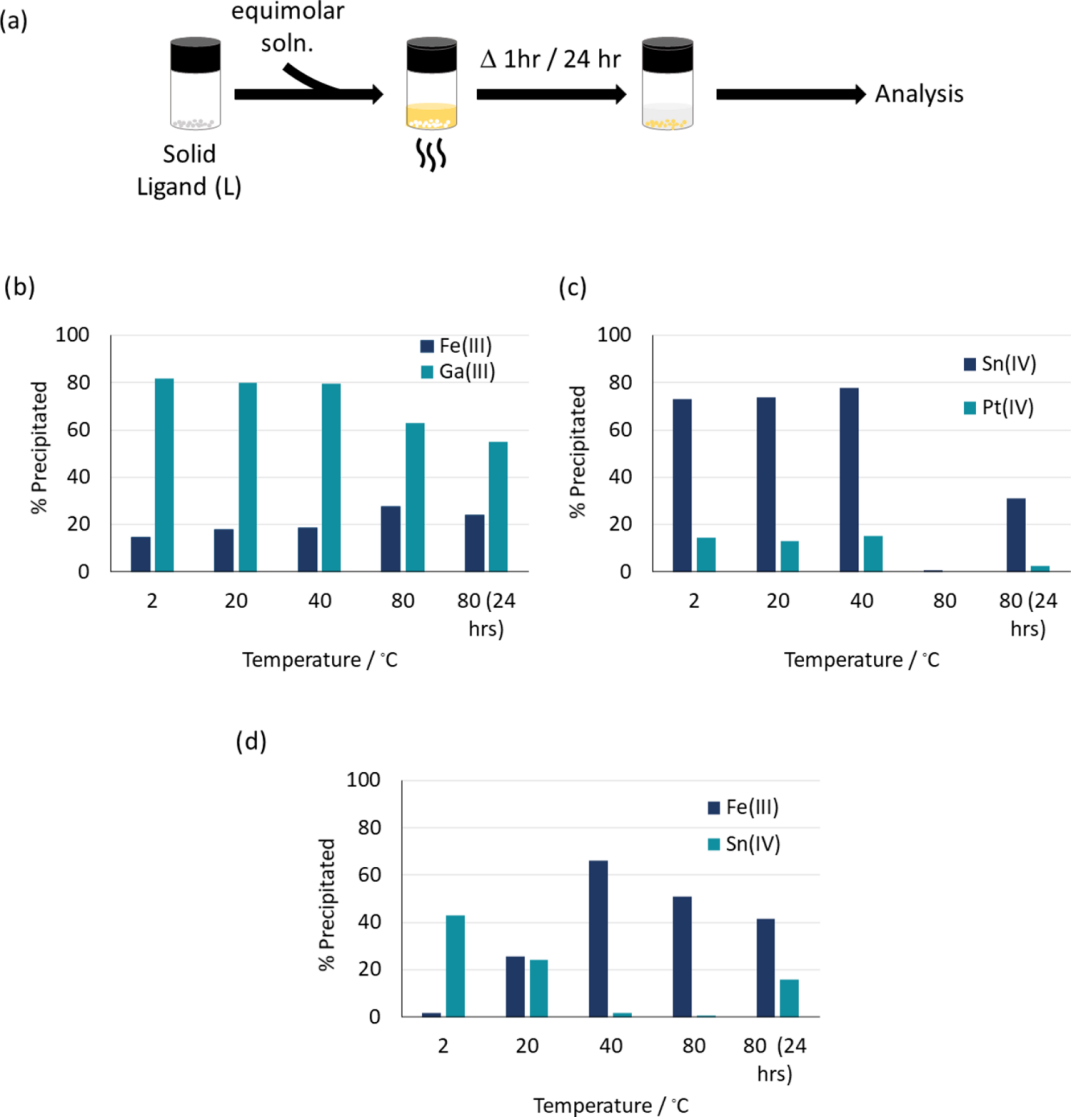
(a) Schematic of competition experiments and results showing percentage metal precipitated at various temperatures after 1 h (and 24 h at 80 °C). (b) Fe/Ga, (c) Sn/Pt and (d) Fe/Sn.

Given that no complete order of selectivity was established in our earlier study, we investigate this here by studying the extent of metal recovery through precipitation as a function of temperature and time. Competition experiments for metalates of the same geometry and charge show that, from an equimolar solution of [FeCl_4_]^−^ and [GaCl_4_]^−^ (both monoanionic tetrahedra), the latter is preferentially precipitated at every temperature value (Figure 2b). This suggests that [H**L**][GaCl_4_] is more thermodynamically stable than [H**L**][FeCl_4_]. Similarly, from an equimolar solution of [SnCl_6_]^2–^ and [PtCl_6_]^2–^ (both dianionic octahedra) selectivity for the former is observed across the whole temperature range (Figure 2c). A final competition experiment between [FeCl_4_]^−^ (least favorable tetrahedral metalate) and [SnCl_6_]^2–^ (most favorable octahedral metalate) was conducted to establish the selectivity order between the tetrahedral and octahedral metalates. In this case, the lower temperature favors precipitation of the dianionic metalate as [H**L**]_2_[SnCl_6_](H_2_O)_2_ while higher temperatures favor [H**L**][FeCl_4_] precipitation (Figure 2d), suggesting that the former is the kinetic product and latter is the thermodynamic product. No selectivity bias was observed at room temperature. In addition, the higher temperature precipitation experiments showed that the precipitation of [FeCl_4_]^−^ was still favored over [SnCl_6_]^2–^. However, after 24 h at 80 °C the quantity of the latter appeared to increase, which was unexpected if this is the kinetic product. A potential explanation is that the nature of the tin-containing precipitate at high temperature has changed, from [H**L**]_2_[SnCl_6_](H_2_O)_2_ to [H**L**]_2_[SnCl_6_]. Subsequent variable temperature combined powder X-ray diffraction (PXRD) and differential scanning calorimetry (DSC), along with single-crystal X-ray diffraction measurements (all documented below) indicated that this is a stable structure formed after heating.

Taken together, these data therefore suggest that the thermodynamic ordering for metalate precipitation, as derived from the variable temperature and time experiments, is as follows (1):
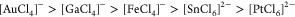
1Thus, it appears that the singly charged, square-planar metalate is precipitated by **L** over a singly charged tetrahedral metalate. While dianonic metalates can be precipitated under more forcing conditions this only occurs for the larger, more charge diffuse octahedral geometry.

To confirm the identity of the phases formed after metal uptake, PXRD data were collected on the precipitates. The PXRD data sets were compared to theoretical PXRD patterns, calculated from the structures determined from single-crystal XRD data collected previously on crystals obtained from the noncompetitive single-metal experiments^46^ and the structure obtained for [H**L**][GaCl_4_] in this study (see Figure S1). The PXRD data sets were fitted using a Pawley refinement routine to refine the unit cell parameters. The refined unit cell parameters and observed, calculated and difference profiles are presented in Figures S2–S7 and confirm the bulk powders have the same structures as the single crystals of each of the metalates. This provides confirmation that the crystal structures can be used as accurate representations of the bulk precipitate structures in the computational modeling study (see below). Furthermore, the presence of water in the tin and platinum precipitates suggests the Hofmeister bias is applicable to selective precipitation methods as well as solvent extraction processes.^32^

### Heat Cycling of Precipitates

The thermodynamic stability of the precipitates was studied over a 20–200 °C heat cycle using *in situ* DSC–PXRD measurements. The data collected during these experiments are simultaneous DSC plots and PXRD patterns, which allows the phase transitions observed in the DSC data to be directly related to changes in the PXRD pattern (Figures S8–S12). No phase changes were observed for the gold precipitate, [H**L**][AuCl_4_], although expansion of the unit cell parameters is observed as the diffraction peaks shift to lower scattering angles, confirmed by Pawley refinement of the unit cell parameters at various temperatures (see Figure S8). The related DSC trace comprised a solitary exothermic peak at *ca*. 180 °C that corresponds to a loss of crystallinity in the PXRD pattern at this temperature. This event likely results from the decomposition of [AuCl_4_]^−^ to AuCl_3_ accompanied by the release of HCl, with the decomposition temperature matching reports of this transition in the literature.^47^ Analysis of the other monoanionic metalate precipitates, [H**L**][FeCl_4_] and [H**L**][GaCl_4_], show similar results to [H**L**][AuCl_4_] in that no phase changes are observed upon heating and cooling, but shifts in the diffraction peaks to lower scattering angles highlight that some thermal expansion is occurring. Pawley refinement was not possible with this data, however, due to the high signal-to-noise ratio as a consequence of the fast scan speed and the presence of Fe in the case of [H**L**][FeCl_4_]. A diffuse peak is observed on the DSC plots at an onset temperature of *ca.* 130 °C for both precipitates, which likely corresponds to a second-order glass transition, with both precipitates affording solid glasses upon cooling. A complete loss in crystallinity at *ca*. 140 °C for both precipitates was observed. These results confirm that the crystal structures of the precipitated species for the monoanionic metalates are the most thermodynamically stable crystal forms, exhibiting stability until transition to an amorphous glassy-like phase at *ca*. 130 °C.

For the precipitates of the dianionic metalates [H**L**]_2_[SnCl_6_](H_2_O)_2_ and [H**L**]_2_[PtCl_6_](H_2_O)_2_, analysis of the DSC-PXRD data indicate that transitions occur, with [H**L**]_2_[SnCl_6_](H_2_O)_2_ undergoing three and [H**L**]_2_[PtCl_6_](H_2_O)_2_ undergoing two. The first transition observed for the [H**L**]_2_[SnCl_6_](H_2_O)_2_ precipitate corresponds to the loss of the water of crystallization, shown by a small bowing endotherm in the DSC curve and changes in the PXRD pattern at *ca*. 45 °C (Figure S11). A distinct shift in the observed diffraction peaks of the tin precipitate without an overall change of phase is found, suggesting that the water in the structure is channel water and is therefore not vital for crystal formation.^48^ The second transition occurs at *ca*. 127 °C and is assigned to a monotropic solid–solid transition, followed by the endotherm melting point of the ligand at 175 °C.^49^ A highly crystalline phase remains which likely corresponds to a SnCl_*x*_ species.

**Figure 3:**
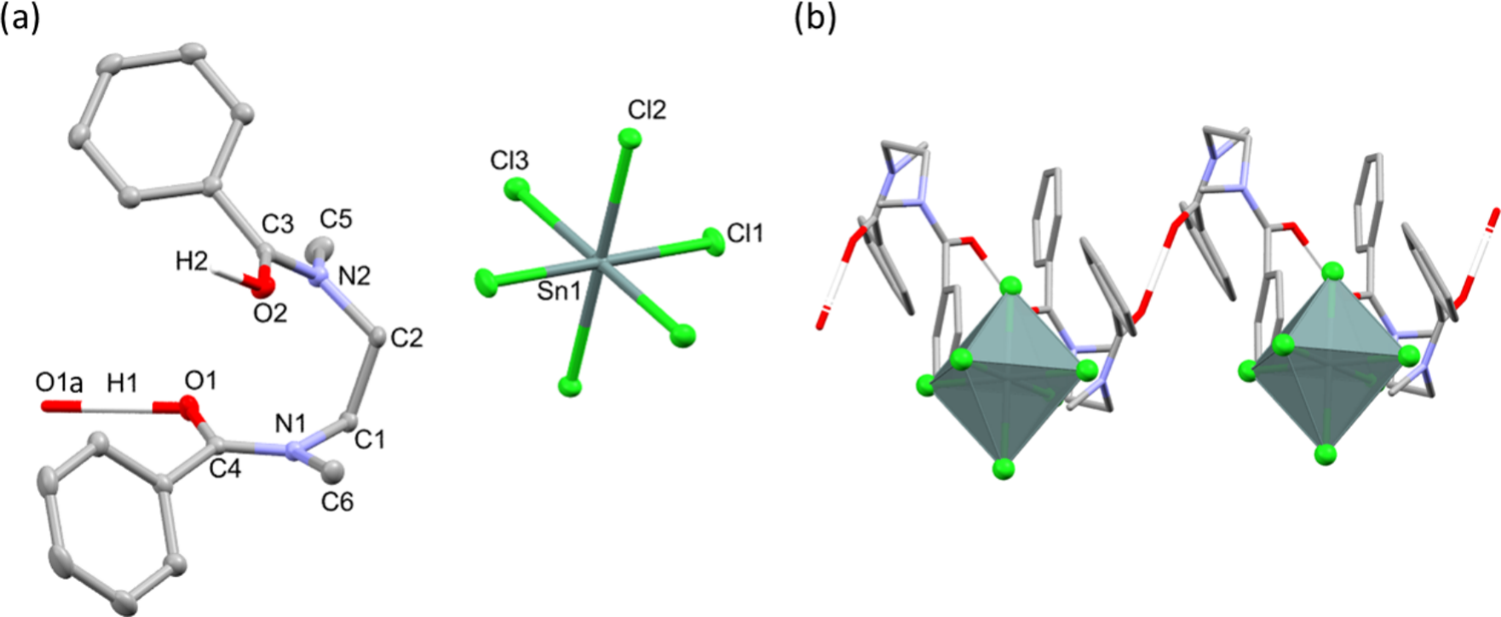
(a) Crystal structure of [H**L**]_2_[SnCl_6_] with hydrogen atoms omitted for clarity except those involved in hydrogen bonding and displacement ellipsoids drawn at 50% probability. (b) Projection of crystal structure along axis coaxial to coil propagation showing the octahedral metalates interleaved between the coil. Atom colors: gray = carbon; red = oxygen; nitrogen = blue; dark green = tin; light green = chlorine; white = hydrogen.

The dehydrated tin phase was further characterized by drying a sample of [H**L**]_2_[SnCl_6_](H_2_O)_2_ at 60 °C and recrystallizing in acetonitrile to afford single crystals. The single-crystal X-ray diffraction data was solved and refined and shows that a new phase has formed through loss of the water (Figure 3a). This is further supported by Pawley refinement of the PXRD pattern for the dehydrated precipitate which shows that the structure of the bulk powder matches that of the single crystal (Figure S6). The crystal structure of the dehydrated phase is very similar to that of the hydrated phase, with only minimal variation in ligand conformation (N1–C1–C2–N2 torsional angle = 78.06(14)° and O1(H1)···O1a = 2.438(1) Å compared with N–C–C–N torsional angle = 80.0(3)° and O1(H1)···O1a = 2.444(3) Å), confirming that the water of crystallization is not an integral component in providing overall crystal stability. A small reduction in unit cell volume (5.8%) is observed, which is expected upon loss of water. The crystal packing remains the same in the new dehydrated phase, with the chloridometalate slotting into grooves between the ligand coils (Figure 3b).

As with the tin precipitate, the first transition observed for [H**L**]_2_[PtCl_6_](H_2_O)_2_ corresponds to the loss of the water of crystallization, denoted by a small bowing endotherm in the DSC at *ca*. 60 °C, with a gradual loss in crystallinity observed in the PXRD pattern from this temperature. The final transition occurs at *ca*. 150 °C, which corresponds to the sample melting. Upon cooling an amorphous solid is obtained. The phase changes observed for the platinum precipitate suggest that water is important in crystal formation for this complex. Given platinum is the least favored metalate, this suggests the presence of water in the crystal is not optimal, likely due to the water inhibiting metalate···ligand interactions (see computational analysis below).

### Computational Modeling

With the order of metalate selectivity established, a full computational analysis of the solid-state structures was conducted in order to rationalize the observed experimental results. The crystal structures were optimized (atoms only, see computational details), and the experimental and computational geometric data were compared (Table 1). Good agreement between both sets of data is seen which confirms the suitability of the computational model. Furthermore, the summary of geometric data highlights the substantial conformational differences exhibited by **L** for the different precipitates, with a significant difference in the bridging N–C–C–N torsional angle observed for the gold complex (56°) compared with all other complexes (80–90°). The magnitude of this torsional angle also appears to be inversely proportional to the length of the repeat unit of the ligand coil, with the longest repeat unit noted in the gold structure, suggesting a relatively decompressed coil, while the other crystal structures present a more compact arrangement. Minimal variation in geometrical data is apparent for the structures of metalates of similar geometry.

**Table 1:** Experimental and Computational Geometric Data for the Ligand Coil Conformations in Each Precipitate, in accordance with Figure 1a,b

	[HL]^+^ length, Å		O–H···O length, Å		C–O–H angle, deg		N–C–C–N torsion, deg		O–C-ring torsion, deg		H–O–C–N torsion, deg	
complex	comp	exp	comp	exp	comp	exp	comp	exp	comp	exp	comp	exp
[H**L**][AuCl_4_]	6.04	6.04	2.41	2.42	117	117	56	55	46	46	158	159
[H**L**][GaCl_4_]	4.34	4.10	2.42	2.44	118	113	85	84	52	49	172	171
[H**L**][FeCl_4_]	4.22	4.35	2.43	2.44	120	118	89	88	51	48	175	180
[H**L**]_2_[SnCl_6_](H_2_O)_2_	4.39	4.23	2.41	2.44	119	118	80	80	57	50	177	177
[H**L**]_2_ [PtCl_6_](H_2_O)_2_	4.38	4.22	2.41	2.44	118	118	81	81	56	50	176	176

**Figure 4:**
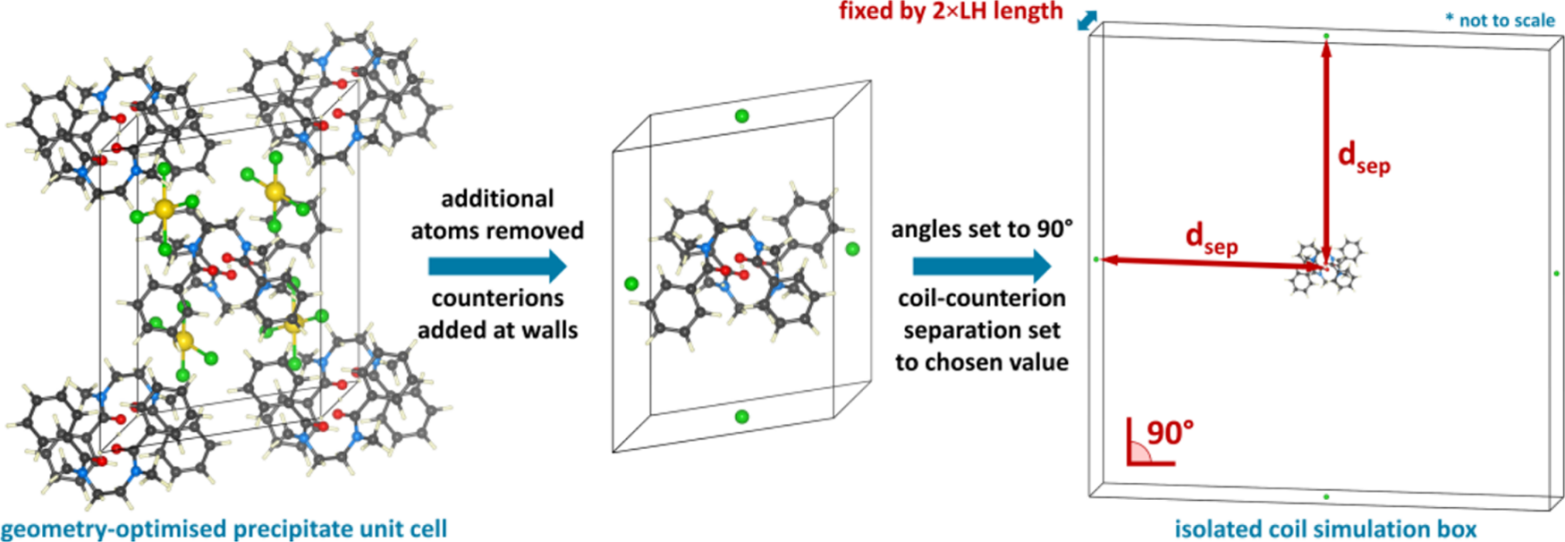
Schematic detailing the construction of the simulation boxes for determining relative ligand strain including the vacuum layer *d*_sep_. Atom colors: gray = carbon, white = hydrogen, blue = nitrogen, red = oxygen, green = chlorine and yellow = gold.

Given the notable differences observed in coil conformation across the different crystal structures, ligand strain was investigated as the first thermodynamic driver to influence metalate selectivity. This aspect could be calculated by comparing the relative energies of the different coil conformations extracted from the optimized crystal structures. However, as the coil is periodic in one dimension, with each repeat unit (2 × [H**L**]^+^) carrying a 2+ charge, construction of a simulation box with counterions (chosen as Cl^–^) placed on the box faces to render the system overall charge neutral was required (Figure 4). A vacuum region was imposed to minimize the electrostatic interactions by uniformly increasing the box size in two dimensions (and therefore the cation/anion separation distance, *d*_sep_). Through a series of single-point energy calculations, it was established that *d*_sep_= 35 Å is sufficient to converge the total energy and still give a computationally accessible model (Figure S13). This distance parameter (*d*_sep_) was kept fixed across all simulations to ensure basis set consistency. Therefore, the only variation between the simulation boxes was the unit cell length coaxial to the ligand coil, which had to be fixed to the length of the coil repeat unit. The energies obtained for the resulting isolated [HL]^+^ simulation box with neutralizing Cl^–^ counterions (as given in Figure 4) are presented in Table 2 as the relative uncorrected coil energies. However, constructing the unit cell in this way affects the distance (and therefore the repulsion) between the chloride counterions as it depends on the 2 × [HL]^+^ repeat length. To correct for this, an additional unit cell was constructed with the ligand dication removed and one of the chloride counterions replaced with a sodium ion; this equates to the energy of two infinite chains of counterions separated by the coil repeat unit length, and thus provides a correction for the counterion repulsion term fixed at the different coil lengths. These correction terms are reported as the relative counterion correction terms in Table 2; adding these corrections to the uncorrected coil energies gives the relative corrected strain energies also reported in Table 2.

**Table 2:** Relative Strain Energies for the [H**L**]^+^ Coils Extracted from the Optimized Crystal Structures, Quoted Per [H**L**]^+^ Molecule, and Ion Exchange Energies Calculated According to Eqs 2 and 3

complex	repeat unit length, Å	relative uncorrected coil energy, kJ mol^–1^	relative counterion correction, kJ mol^–1^	relative corrected strain energy, kJ mol^–1^	Δ*U*_ex_, kJ mol^–1^
[H**L**][AuCl_4_]	12.08	0.00	0.00	0.00	0.00
[H**L**][GaCl_4_]	8.67	2.59	–7.53	–4.94	18.93
[H**L**][FeCl_4_]	8.63	2.71	–7.65	–4.94	34.09
[H**L**]_2_[SnCl_6_](H_2_O)_2_	8.77	5.53	–7.28	–1.93	104.51
[H**L**]_2_[PtCl_6_](H_2_O)_2_	8.76	6.24	–7.31	–1.07	120.78

The results of these calculations (Table 2) show that the variation in ligand strain energy for the different compounds is low (<5 kJ mol^–1^) and, moreover, the stretched coil seen in the [H**L**][AuCl_4_] structure is the least stable configuration. It is therefore highly unlikely that coil strain is the primary driver for metalate selectivity. Furthermore, if the process is thermodynamically driven as suggested by the experimental study, it also indicates that this less favorable configuration must support stronger intermolecular interactions in the crystal lattice.^45^

**Figure 5:**
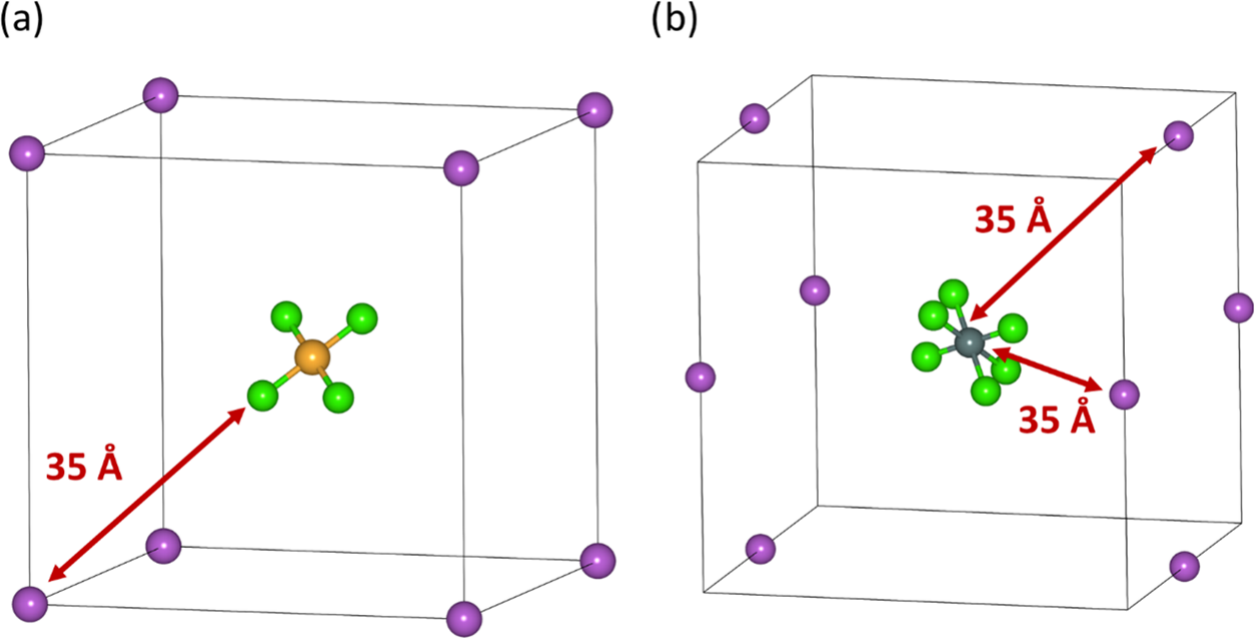
Simulation boxes used to approximate the energies of isolated metalates, centered in cubic unit cells and charge-balanced by (a) one or (b) two Na^+^ counterions, separated by a distance of 35 Å. Atom colors: yellow = gold, light green = chlorine, purple = sodium, and dark green = tin.

As such, the differences in intermolecular interaction energies in the crystal structures of the metalate series were quantified. As the structures incorporate different metalate ions, energies relative to the [H**L**][AuCl_4_] structure were calculated using ion exchange eqs 2 and 3, where M = Ga, Fe in eq 2, and Sn, Pt in eq 3.
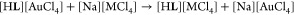
2
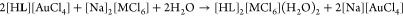
3In this way, the energy required to theoretically displace [AuCl_4_]^−^ from the [H**L**]_*x*_[MCl_*y*_] structure by another metalate in the solid state can be determined, providing an indication of the relative stabilities of the precipitated compounds. While ion exchange reactions are routinely employed to obtain complex stability data in isolated-molecule calculations,^50−52^ it is rare to apply them to solid-state structures. The challenge it presents is that all species named in the equations must have known structures. Unfortunately, crystal structures for equivalent metalate salts, namely, [Na][MCl_4_] and [Na]_2_[MCl_6_], are unavailable, but, as relative energies are being calculated, artificial unit cells can be constructed for these structures, analogous to the procedure adopted to calculate the energies of the isolated coils. In this case, each metalate was placed in the middle of a cubic simulation box with the sodium counterion placed either in the corner (for [MCl_4_]^−^) or edges (for [MCl_6_]^2–^) of the cube (Figure 5). The crystal structure of hexagonal ice was used as a model for the structures containing water of crystallization.

The results of these calculations (Table 2, see also Tables S1 and S2 for further details) show that [H**L**][AuCl_4_] is the most thermodynamically stable structure, as all calculated anion exchange energies (Δ*U*_ex_) are positive values. The differences in energy are larger than those calculated for ligand strain and, furthermore, afford the correct order of metalate selectivity according to the competition experiments. This supports the hypothesis that the flexible ligand coil adopts the conformation that maximizes the strength of the intermolecular interactions. Caution must be stressed, however, in overinterpreting the comparison between the [MCl_4_]^−^ and [MCl_6_]^2–^ exchange energies, as the computational procedure to determine Δ*U*_ex_ differs for the two anion types (eqs 2 and 3, respectively). Errors will likely arise due to differences in the counterion positions in the [Na][MCl_4_] and [Na]_2_[MCl_6_] calculations, and also due to the need to account for the presence of water molecules in the tin and platinum structures. However, the prediction that [H**L**]_2_[SnCl_6_](H_2_O)_2_ presents more stable intermolecular interactions than [H**L**]_2_[PtCl_6_](H_2_O)_2_ holds.

The Δ*U*_ex_ values (Table 2) are based on the internal energies and therefore overlook the contributions from the zero-point energy (ZPE) and entropy (*S*) terms. Given that the predicted Δ*U*_ex_ values are small, it is possible that neglecting the thermodynamic corrections may result in significant error. To account for this, corrections for the [AuCl_4_]^−^, [GaCl_4_]^−^ exchange reaction were calculated to recast Δ*U*_ex_ as Δ*G*_ex_ (Table S2) for which the large positive value of Δ*G*_ex_ confirms that anion exchange is disfavored on both enthalpic and entropic grounds.

**Figure 6:**
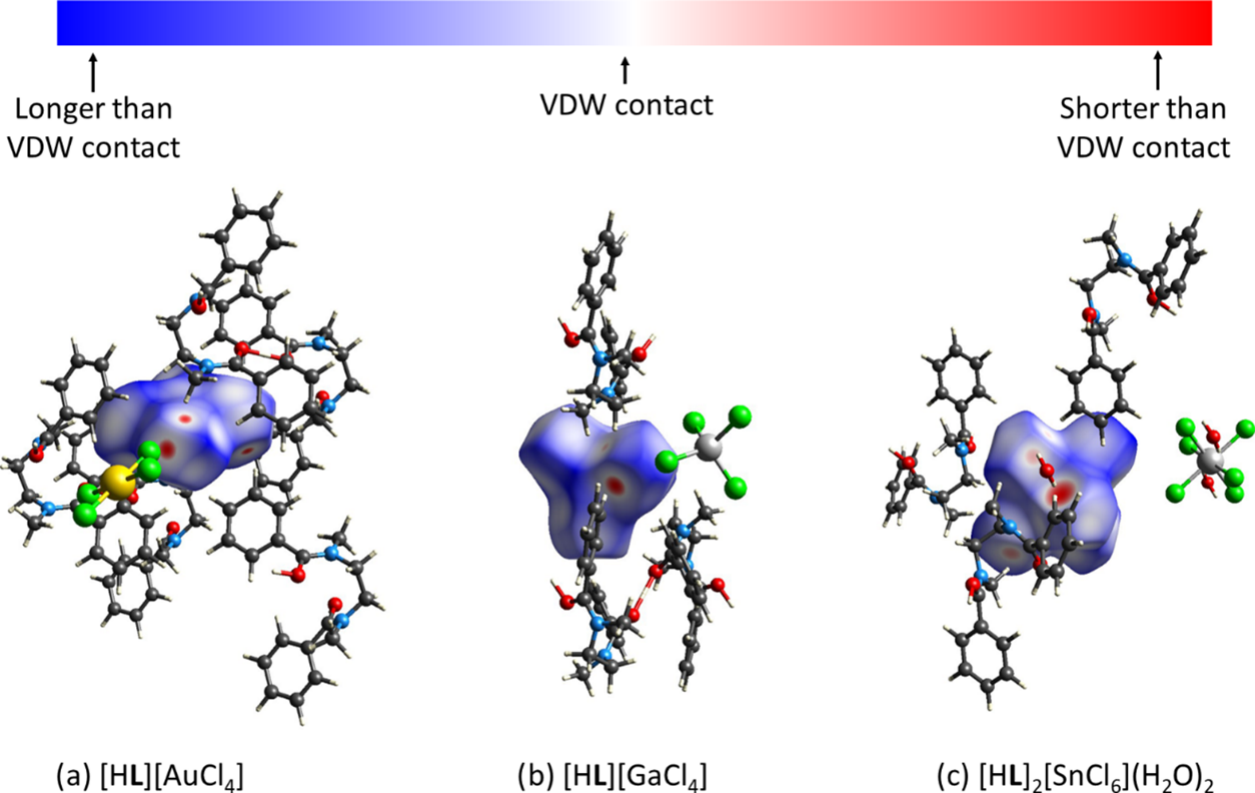
Normalized contact distance, *d*_norm_, mapped on the Hirshfeld surfaces generated with CrystalExplorer for (a) [H**L**][AuCl_4_], (b) [H**L**][GaCl_4_], and (c) [H**L**]_2_[SnCl_6_](H_2_O)_2_. Close contacts are shown in red. Atom colors: gray = carbon, white = hydrogen, blue = nitrogen, red = oxygen, yellow = gold, green = chlorine, silver = gallium (b) or tin (c).

With the experimental order of selectivity rationalized by the computational modeling, an in-depth analysis of the intermolecular interactions within the crystal structures was undertaken, in order to rationalize the stability ordering observed. Multiple computational methods were employed to give a cohesive and complete view of these interactions. Hirshfeld surface analysis was conducted first, to identify key areas of interest and potential intermolecular bonding sites. Here, the contact surface defined between neighboring molecules is analyzed in terms of the normalized contact distance *d*_norm_, which differentiates between regions where the nearest atoms are in closer contact than the sum of their van der Waals (VDW) radii from those where they are further apart. The *d*_norm_ maps for the gold, gallium, and tin precipitates, representing ligand complexation to a square-planar, tetrahedral, and octahedral complex, respectively (Figure 6), highlight the nonclassical hydrogen bonding interactions between the ligand and metalates, as identified previously by noncovalent interaction (NCI) plots as major interactions present in all structures.^46^ A significant interaction is observed in the gold structure, namely a close contact between the chlorine atoms of neighboring metalates. This interaction is not apparent in the gallium or tin structures, suggesting it may be significant in metalate selectivity. Aside from the nonclassical hydrogen bonds, no other significant interactions are detected in the gallium structure. For the tin structure, strong interactions are observed between the bound water molecules and the metalate. The Hirshfeld surfaces were further interrogated to generate fragment patches, where the surface area is divided into regions based on the identity of the interacting neighboring molecules. In this way, the coordination numbers of the different crystallographic components, along with the percentage of the Hirshfeld surface involved in specific intermolecular interactions, can be deduced. This analysis reveals that, on average, **L** displays a greater surface area contact on interacting with [AuCl_4_]^−^ compared with the other metalates (Figure S14), which suggests superior encapsulation of the gold metalate by **L**.

**Figure 7:**
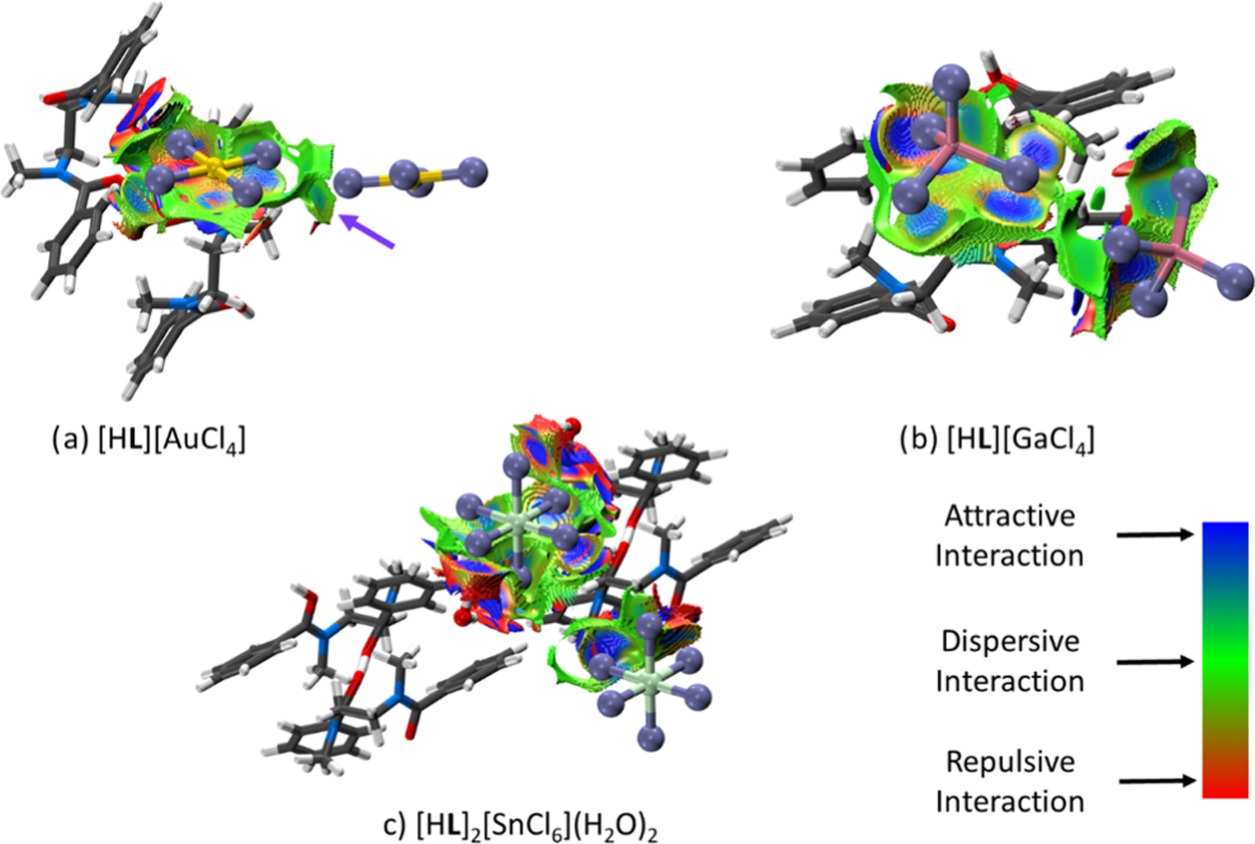
*s*-Isosurface plots (*s* = 3.6) for selected fragments from (a) [H**L**][AuCl_4_], (b) [H**L**][GaCl_4_], and (c) [H**L**]_2_[SnCl_6_](H_2_O)_2_ crystal structures derived from the NCI analysis. Attractive interactions are shown in blue, dispersive in green, and repulsive in red. Note the red regions around the edges of the plots are artifacts of the core augmentation procedure employed to account for the missing core electrons in the valence-only density files. Atom colors: gray = carbon, white = hydrogen, blue = nitrogen, red = oxygen, yellow = gold, purple = chlorine, pink = gallium, light green = tin.

The intermolecular interactions identified through the Hirshfeld surface analysis were further probed using noncovalent interaction (NCI) plots and quantum theory of atoms in molecules (QTAIM) analysis.^53−55^ Although NCI plots were reported in our previous work, a more in-depth study was undertaken here that explores a wider sphere of interactions beyond the immediate metalate···**L** interaction. The three-dimensional *s*-isosurface plots for the [H**L**][AuCl_4_], [H**L**][GaCl_4_], and [H**L**]_2_[SnCl_6_](H_2_O)_2_ complexes (Figure 7) show all metalates to be encapsulated by **L**, with a large number of dispersive interactions (shown in green) present. Attractive interactions (blue) are observed in all plots, with nonclassical hydrogen bonding between CH_1–3_ groups and the metalates comprising the majority of these interactions. Repulsive interactions (red) occur where the oxygen and nitrogen atoms on the ligand interact with the metalate, or where oxygen atoms are proximate. Notably, a unique [H**L**]^+^···metal interaction is observed in the gold complex in which two CH_2_ groups of **L** are positioned axially to the gold center and which facilitates complete encapsulation of the metalate. This direct interaction with the metal center is not observed in any of the other structures, presumably due to their nonplanar geometries. This is therefore likely to be a significant interaction that contributes to the enhanced stability of the [H**L**][AuCl_4_] structure. Furthermore, the [AuCl_4_]^−^···[AuCl_4_]^−^ interaction seen above in the Hirshfeld surface analysis is also described in the NCI plot as an attractive interaction (the green/blue disk in Figure 7a marked by the purple arrow). A similar, but weaker, interaction is observed in the NCI plot of the gallium complex [H**L**][GaCl_4_], while it is completely absent in the NCI plot of the tin complex [H**L**]_2_[SnCl_6_](H_2_O)_2_, further consolidating the importance of this interaction. The [SnCl_6_]^2–^···H_2_O interaction detected in the Hirshfeld surface analysis is also present in the NCI plot as a strong attractive interaction (blue disk).

The NCI 2D reduced density gradient (*s*) vs sign(λ_2_)ρ plots for the [H**L**][AuCl_4_], [H**L**][GaCl_4_], [H**L**][FeCl_4_] and [H**L**][SnCl_6_](H_2_O)_2_ crystal structures (Figure S15) give further insight into the nature of the intermolecular interactions. Minimal variation is observed between the strong interactions (hydrogen bonds and [H**L**]^+^···[H**L**]^+^) across the series, excluding the [MCl_6_]^2–^···H_2_O interaction only present for [H**L**]_2_[SnCl_6_](H_2_O)_2_. Therefore, analysis of the dispersive interactions is required to characterize the [H**L**]^+^···[MCl_*x*_]^*y*−^ and [MCl_*x*_]^*y*−^···[MCl_*x*_]^*y*−^ interactions, which are the most important structure-directing interactions. The most notable difference across the plots is the apparent increase in the strength of the [H**L**]^+^···[MCl_*x*_]^*y*−^ interactions with the reverse order of selectivity, i.e., the interactions appear weakest in the [H**L**][AuCl_4_] plot. However, this arises as the multiplicities of the individual interactions have not been taken into account and therefore this does not reflect the overall strength of host···guest interactions.

To better account for this, a QTAIM study was undertaken to provide a complementary quantitative description of the intermolecular interactions observed in the NCI plots. For each structure, the densities of the bond critical points (BCPs) are normalized against the density values obtained for a benchmarked hydrogen bond BCP common to all structures (Table 3). This step was taken as this interaction should be of equal strength in all structures, based on its signature in the NCI plots and the similar hydrogen bond lengths observed across the complexes (see Table 1). This was necessary as the densities at the BCPs cannot be quoted directly as the evaluation of the electron densities is susceptible to the completeness of the critical point list and to the grid basis, which varies across the structures due to different unit cell vectors. However, by benchmarking the calculated densities against a uniform interaction common to all crystal structures, comparisons between them can be made.

**Table 3:** Electron Density Fractions at the BCPs Found in the [H**L**][AuCl_4_], [H**L**][GaCl_4_], [H**L**][FeCl_4_], [H**L**]_2_[SnCl_6_](H_2_O)_2_, and [PtCl_6_][H**L**]_2_(H_2_O)_2_ Crystal Structures by QTAIM Analysis^a^

	complex				
interaction	[AuCl_4_]^−^	[GaCl_4_]^−^	[FeCl_4_]^−^	[SnCl_6_]^2–^	[PtCl_6_]^2–^
	Electron Density at BCP, electrons Å^–3^				
benchmark hydrogen bond	6.38	10.2	10.3	9.34	9.18
	Fraction of Hydrogen Bond Density, %				
[MCl_4_]^−^···[MCl_4_]^−^	5.06 (×2)	1.81 (×2)	2.18		
			1.42		
subtotal	10.11	3.63	3.60		
CH_3_···[MCl_*x*_]^*y*−^	5.51 (×2)	5.32	4.62	6.84 (×2)	7.38 (×2)
	2.91 (×2)	3.99	3.80	3.50 (×2)	3.36 (×2)
	2.56 (×2)	3.36	3.27	0.86 (×2)	0.91 (×2)
		2.72	2.99		
		1.61	2.09		
		1.22	1.63		
subtotal	21.97	18.22	18.40	22.41	23.31
CH_2_···[MCl_*x*_]^*y*−^	3.11	4.77	5.41	4.83 (×2)	4.79 (×2)
	3.12	3.36	3.83	4.54 (×2)	4.72 (×2)
		3.19	2.84	4.34 (×2)	4.60 (×2)
		2.83	2.38	3.79 (×2)	3.68 (×2)
		2.53	1.40	3.18 (×2)	3.63 (×2)
				1.58 (×2)	1.98 (×2)
subtotal	6.23	16.67	15.85	44.53	46.79
Ar–H···[MCl_*x*_]^*y*−^	5.29 (×2)	7.25	6.50	5.02 (×2)	4.58 (×2)
	3.92 (×2)	4.90	4.25	4.62 (×2)	4.52 (×2)
	3.90 (×2)	4.73	4.01	4.09 (×2)	4.13 (×2)
	3.64 (×2)	4.03	3.92	3.91 (×2)	3.94 (×2)
	3.59 (×2)	3.84	3.17	3.00 (×2)	2.98 (×2)
	3.28 (×2)	3.42 (×2)	3.02	2.25 (×2)	2.46 (×2)
	3.22 (×2)	3.22	2.97 (×2)	2.24 (×2)	2.28 (×2)
	2.21 (×2)	3.06	2.60	2.19 (×2)	2.10 (×2)
		2.90	2.57	1.96 (×2)	1.98 (×2)
		2.70	2.46	1.25 (×2)	1.25 (×2)
		2.69	2.31	1.01	1.04 (×2)
		2.54	2.13	1.00	
		2.22	1.80		
		1.99	1.33		
		1.25	0.83		
subtotal	58.12	54.14	46.84	63.04	62.55
H_2_O···[MCl_*x*_]^*y*−^	n/a	n/a	n/a	14.18 (×2)	14.67 (×2)
				5.90 (×2)	6.16 (×2)
subtotal				40.17	41.66
O···[MCl_*x*_]^*y*−^	3.29 (×2)				
	1.79				
subtotal	8.37				
N···[MCl_*x*_]^*y*−^		2.06			
sum of all density fractions	104.79	94.72	84.70	170.16	174.31

a Values are normalized against the electron densities of the coil hydrogen bonds to draw comparisons across the series (see text). Where BCPs are replicated by symmetry, this is denoted as “×2”.

Turning first to the sum of the electron densities recorded at the BCPs, while the density fractions appear to be significantly larger in the tin and platinum structures this arises due to the presence of two [H**L**]^+^ units for every metalate in the crystal structure; when these totals are halved to give the number of BCPs on a per ligand basis, the results across the whole data set matches the experimentally determined order of metalate selectivity. This strongly suggests that it is the strength of the intermolecular interactions that dictates the selectivity ordering in a competitive mixed-metal environment which concurs with earlier observations that the process is thermodynamically driven.

**Figure 8:**
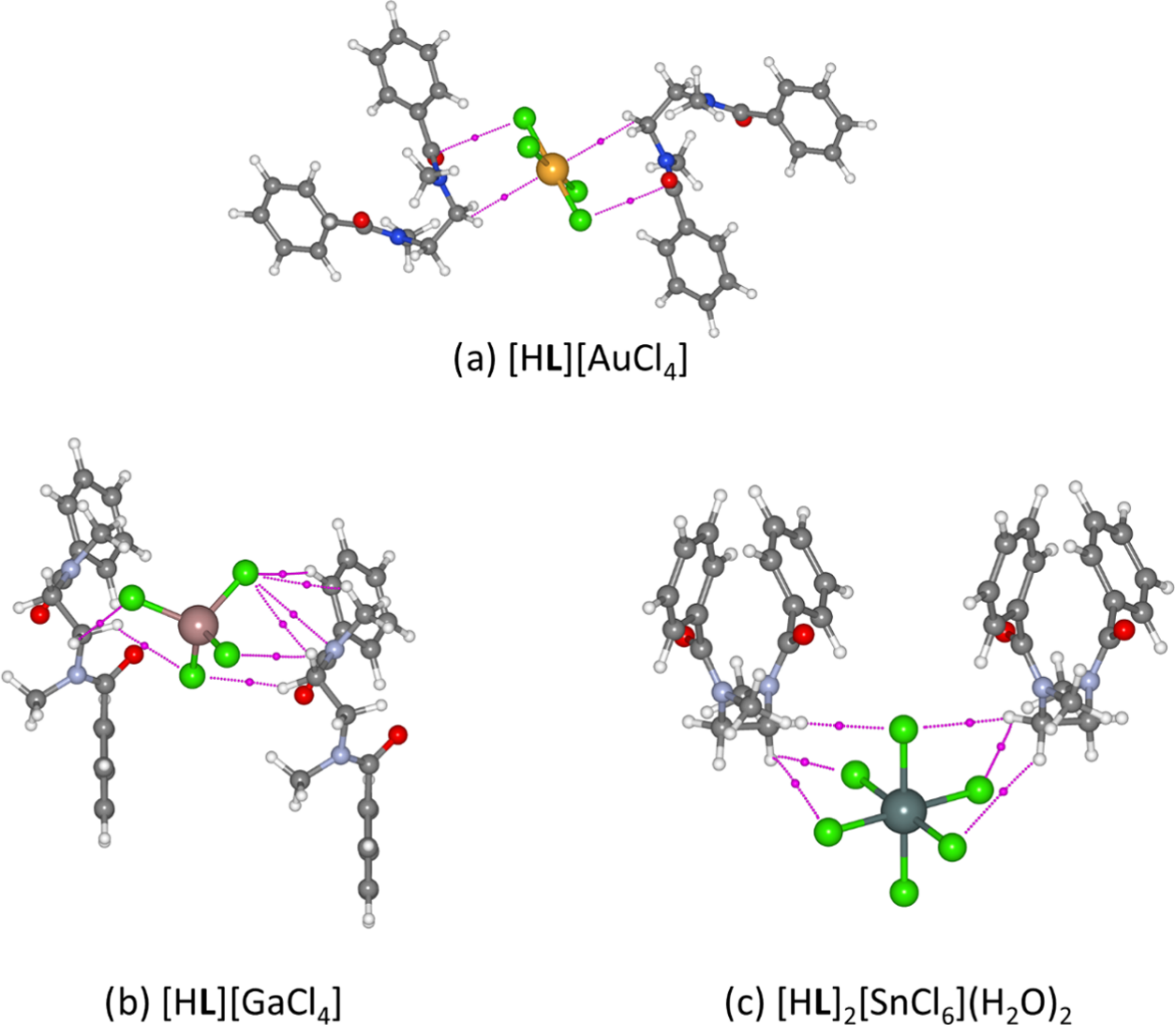
Bond paths and critical points for the CH_2_···[MCl_*x*_]^y–^ interactions in (a) [H**L**][AuCl_4_] (3.40 Å), (b) [H**L**][GaCl_4_] (3.00, 3.12, 2.81 Å), and (c) [H**L**]_2_[SnCl_6_](H_2_O)_2_ (3.00, 3.39, 3.67 Å) generated from the QTAIM analysis. For the tin structure, only the symmetry inequivalent bond paths and BCPs are shown. Atom colors: gray = carbon, white = hydrogen, blue = nitrogen, red = oxygen, yellow = gold, green = chlorine, light green = gallium, and light purple = tin.

**Figure 9:**
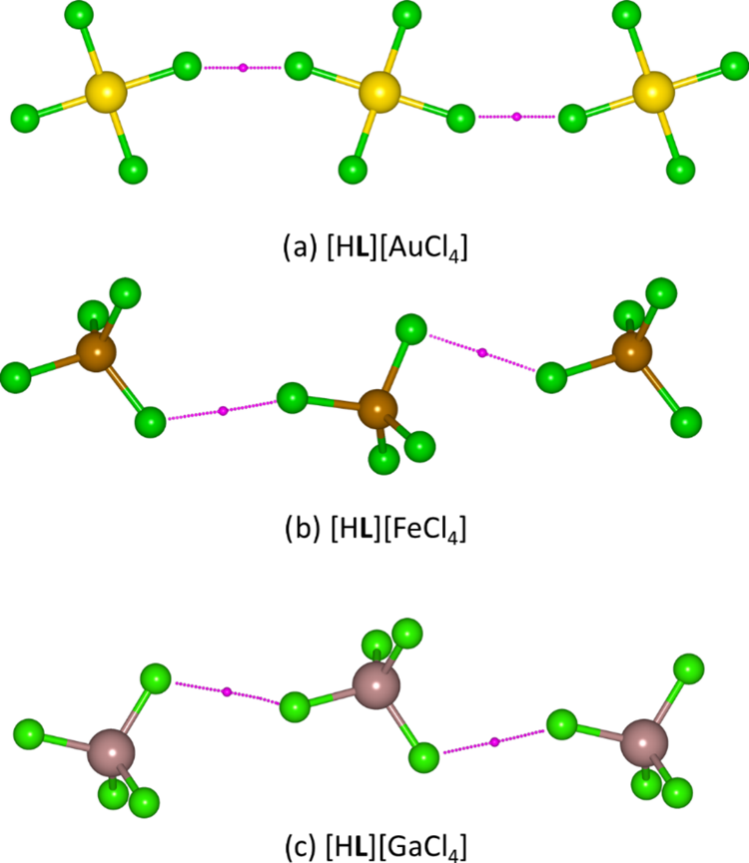
Molecular graphs for [MCl_*x*_]^*y*−^···[MCl_*x*_]^y–^ interactions obtained from QTAIM analysis for (a) [H**L**][AuCl_4_] (Cl···Cl = 3.36 Å, Au–Cl–Cl 147°), (b) [H**L**][FeCl_4_] (Cl···Cl = 3.87 and 4.09 Å, Fe–Cl···Cl = 145°, Cl···Cl–Fe = 97°, Fe–Cl···Cl = 160° and Cl···Cl–Fe = 115°), and (c) [H**L**][GaCl_4_] (Cl···Cl = 3.95 Å, Ga–Cl···Cl = 154°, Cl···Cl–Ga = 110°). Atom colors: yellow = gold, green = chlorine, brown = iron, and dark pink = gallium.

The QTAIM analysis highlights that special attention to the CH_2_···[MCl_*x*_]^*y*−^ and [MCl_*x*_]^y–^···[MCl_*x*_]^*y*−^ interactions is warranted, as these vary substantially in strength across the series. The bond paths found in [H**L**][AuCl_4_] (Figure 8) show that, as in the NCI study, the CH_2_ groups directly interact with the metal center, to create pseudo-anagostic C–H···Au interactions where the gold acts as a nucleophile. These interactions are of similar strength to both aurophilic interactions,^56^ and anagostic C–H···Au interactions.^57^ While this interaction is one of the weakest in terms of BCP density, the direct interaction of **L** with the gold center results in complete encapsulation of the metalate and, by extension, increased stability of the structure. In all other structures, [H**L**]^+^ interacts with the corners, edges, or faces of the metalate structures in a more standard fashion.^58^ The molecular graphs of the [MCl_*x*_]^*y*−^···[MCl_*x*_]^*y*−^ interactions (Figure 9) show that the angles of contact between the metalates and bond path lengths correspond to two well-documented types of halogen···halogen interaction:^59^ a symmetric type-I interaction in the gold structure (where θ_1_ = θ_2_); bent type-II interactions in the gallium and iron structures (where θ_1_ ≈ 180° and θ_2_ ≈ 90°). These interactions have been reported to be both stabilizing and structure-directing,^60−62^ with type-I generally characterized by shorter interhalogen separations and the ability to reduce repulsion by interfacing the neutral regions on the electrostatic potential surfaces of the interlacing atoms.^63^ Conversely, type-II interactions arise due to the attraction between electrophilic and nucleophilic regions on the interacting halogens.^64^ From the BCPs located in the QTAIM study, stronger type-I interactions were found in the [H**L**][AuCl_4_] structure, with similar Cl···Cl contacts to those reported in the literature.^65^ The weaker type-II interactions were found in [H**L**][GaCl_4_] and [H**L**][FeCl_4_] while the interaction was absent altogether in [H**L**]_2_[SnCl_6_](H_2_O)_2_ and [H**L**]_2_[PtCl_6_](H_2_O)_2_. This also maps the observed experimental results, suggesting that Cl···Cl interactions are an important factor in directing metalate selectivity ordering.

## Conclusions

The simple diamide ligand **L** acts as a selective precipitant that can recover gold from mixed-metal acidic solutions typical of e-waste leach streams, but until now the reason for this selectivity was unknown. We have addressed this here through a combined experimental and computational modeling study. Direct competition experiments and analysis of the thermal stability of the metal-containing precipitates showed that thermodynamic stability is an important driver for selectivity. The computational modeling has shown that the process is driven by the strength of the intermolecular interactions, and in particular has highlighted the importance of the type-I halogen···halogen bonding interaction in [H**L**][AuCl_4_] and the pseudo-anagostic C–H···Au interaction that provides enhanced stability in the solid-state structure. This holistic experimental and computational approach provides a complete and fundamental view of the factors that underpin precipitate stability in relation to the selective separation of metals by **L**, and provides a baseline for the application of rational ligand design in providing solutions to the modern-day hydrometallurgical separations.

## Experimental and Computational Methods

All solvents and reagents were used as received from Sigma-Aldrich, Fisher Scientific UK, Alfa Aesar, Acros Organics, or VWR International. Ultrapure water was obtained from a Milli-Q purification system.

### Preparation of *N*,*N*′-1,2-Ethanediylbis(*N*-methylbenzamide) (**L**)

**L** was prepared according to an adapted method from the literature.^46,66^ A solution of *N*,*N*′-dimethylethylenediamine (1.76 g, 2.15 mL, 20 mmol) in CH_2_Cl_2_ (25 mL) in a two-neck round-bottom flask fitted with an air condenser and Suba seal was cooled in an ice bath. A solution of benzoyl chloride (7.03 g, 5.81 mL, 50 mmol) in CH_2_Cl_2_ (25 mL) was added slowly to the reaction mixture by syringe. The reaction was brought to room temperature and left for 18 h. The reaction mixture was then diluted in CH_2_Cl_2_ (50 mL) and subsequently washed with dilute HCl (3 × 100 mL), ultrapure water (3 × 100 mL), and brine (3 × 100 mL). The organic phase was dried with sodium sulfate, and CH_2_Cl_2_ was removed under vacuum. The resulting off-white solids were recrystallized from hot toluene to yield white crystals of **L** (4.54 g, 77%). ^1^H NMR (500.12 MHz, CDCl_3_): δ = 2.63–3.01 (br m, 6H, NCH_3_), 3.24–4.08 (br m, 4H, NCH_2_), 7.32–7.49 (m, 10H, aromatic CH); ^13^C NMR (125.76 MHz, CDCl_3_): δ = 37.9, 44.5, 126.9, 128.4, 129.5, 136.3, 170 ppm.

### Direct Competition Experiments

Equimolar stock solutions of FeCl_3_/GaCl_3_, SnCl_4_/Na_2_PtCl_6_, and SnCl_4_/FeCl_3_ were prepared in 6 M HCl (0.01 M each). Solid **L** (either 1 mol or 2 mol equiv; **L**/Fe/Ga 1:1:1, 5.6 mg, 0.02 mmol, **L**/Sn/Pt 2:1:1, 11.2 mg, 0.04 mmol, and **L**/Fe/Sn 1:1:1, 5.6 mg, 0.02 mmol) was added to a vial with a magnetic stir bar followed by the mixed-metal solution (2 mL). Samples stirred for 1 h at 500 rpm at room temperature (20 °C). The stir bar was then removed and the solutions were centrifuged to separate solids from the supernatant. This process was then repeated at 2, 40 and 80 °C. Supernatants and feed samples diluted ×100 in 2% nitric acid for analysis by ICP-OES to determine metal content. All experiments were carried out in duplicate.

### ICP-OES Analysis

Analysis of metal content in samples was carried out using ICP-OES on a PerkinElmer Optima 8300 Inductively Coupled Plasma Optical Emission Spectrometer. All samples were diluted in 2% nitric acid and taken up at a rate of 1.3 mL min s^–1^, with the following argon gas flow parameters: 12 L min^–1^ plasma, 0.2 min^–1^ auxiliary, and 0.6 L min^–1^ nebulizer. ICP-OES calibration standards were obtained from VWR International, SCP Science, or Sigma-Aldrich.

### Sample Preparation for Diffraction Measurements

Samples were prepared by contacting solid **L** (0.0296 g, 0.1 mmol) with a solution of either HAuCl_4_, FeCl_3_, GaCl_3_, SnCl_4_, or Na_2_PtCl_6_ in 6 M HCl (2 mL, 0.05 M) and stirring for 1 h (Au, Fe, Ga, Sn) or 24 h. (Pt). The precipitates were filtered under vacuum and allowed to air-dry. To prepare the dehydrated tin powder, the sample was first filtered and then placed in a 60 °C oven for 4 h.

### Single-Crystal X-ray Diffraction

X-ray crystallographic data were collected at 120 K on an Oxford Diffraction Excalibur diffractometer ([H**L**][GaCl_4_]) using graphite monochromated Mo Kα radiation equipped with an Eos CCD detector (λ = 0.71073 Å) or on a Supernova Dual, Cu at home/near Atlas diffractometer ([H**L**]_2_[SnCl_6_]) using Cu Kα radiation (λ = 1.54184 Å). Structures were solved using ShelXT direct methods and refined using a full-matrix least-squares refinement using ShelXL.^67−69^ All programs were used within the Olex suites.^70^

### Powder X-ray Diffraction

Powder X-ray diffraction (PXRD) data of [H**L**][AuCl_4_], [H**L**][FeCl_4_], and [H**L**]_2_[SnCl_6_] were collected at the Diamond Light Source on the high-resolution powder diffraction beamline (I11). Samples were packed into borosilicate capillaries and analyzed using a Si-calibrated wavelength of λ = 0.825970 Å. PXRD data of [H**L**][GaCl_4_], [H**L**]_2_[SnCl_6_](H_2_O)_2_, and [H**L**]_2_[PtCl_6_](H_2_O)_2_ were collected on a Bruker D8 Advance diffractometer in transmission geometry with Cu Kα radiation (λ = 1.5406 Å). Data were collected over the 2θ range of 5–30° or 7–30° for 30 min or 1 h. Data were analyzed, to refine the unit cell parameters, using a Pawley fitting routine, part of the Topas Academic (version 6) software suite.

### Differential Scanning Calorimetry-Coupled Powder X-ray Diffraction

Simultaneous PXRD-DSC data were collected on a Rigaku Smartlab XE powder X-ray diffractometer using the PXRD-DSC sample environment chamber, set up in Bragg–Bretano geometry. Samples were loaded into open aluminum pans for analysis. PXRD data were collected using Cu Kα1 radiation (λ = 1.5401 Å) selected from a Johannsson monochromator and HyPix3000 detector (in 1D scan mode). Incident and receiving soller slits of 2.5° were used and the length limiting slit was 5 mm, with an incident slit of 1/3°. Samples were measured over a 20–200 °C heat cycle, with a heating rate of 1 °C min^–1^ over the 2θ range of 7–31° 2θ, with a step size of 0.01° and a scan speed of 4° min^–1^ (for the tin precipitate the scan speed was 6° min^–1^).

### DFT Energy Calculations

Calculations were performed using CASTEP v18.1,^71^ utilizing the PBE generalized gradient approximation functional^72^ and TS dispersion correction scheme.^73,74^ The basis set was constructed using “on-the-fly” ultrasoft pseudopotentials^75^ coupled to plane-wave basis sets expressed at 900 eV.^76^ Calculations involving [FeCl_4_]^−^ were spin-polarized with an initial number of five unpaired electrons per Fe atom. The electronic energy convergence threshold for self-consistent field (SCF) calculations was set to 1.0 × 10^–9^ eV per atom. The Brillouin zone sampling grid was set to 0.05 Å^–1^. Input precipitate geometries were taken from the Cambridge Crystallographic Data Centre (CCDC) under reference numbers 2084239 ([H**L**][AuCl_4_]), 2084238 ([H**L**][FeCl_4_]), 2084235 ([H**L**]_2_[SnCl_6_](H_2_O)_2_), 2084240 ([H**L**]_2_[PtCl_6_](H_2_O)_2_),^46^ ([H**L**][GaCl_4_]) 2308454. The input geometry for H_2_O was taken from the crystal structure of hexagonal ice (I_h_) acquired from the Inorganic Crystal Structure Database (ICSD) with collection code 27837. Structures were converted to CASTEP *.cell files using the Atomic Simulation Environment (ASE) Python library.^77^ The initial structures were relaxed with fixed unit cell vectors using the LBFGS algorithm^78^ until the energy change between consecutive cycles was below 2.0 × 10^–5^ eV per atom, the forces on all atoms were <0.05 eV Å^–1^, and the maximum displacement was <2.0 × 10^–3^ Å. Geometry-optimized precipitate crystal structures in Figures 1 and 3 were visualized using Mercury 2020.3.0.^79^

To obtain zero-point energy and entropy corrections for [H**L**][AuCl_4_] and [H**L**][GaCl_4_], structures were further optimized with more stringent criteria in order to obtain sufficiently optimized structures for reliable phonon calculations, which were expressed at the Γ-position only.^80^ The energy convergence tolerance in the geometry optimization was reduced to 2.0 × 10^–6^ eV per atom, the force tolerance to 0.005 eV Å^–1^, and the displacement tolerance to 2.0 × 10^–4^ Å. The fine grid scale for SCF calculations was increased to 4.0. The pseudopotentials were switched to norm-conserving PBE OPIUM. To obtain zero-point energy and entropy corrections for [AuCl_4_]^−^ and [GaCl_4_]^−^, the atomic coordinates were reoptimized using Gaussian 16^81^ at the PBE1PBE/Def2TZVP level of theory with vibrational frequency analysis.

To construct simulation boxes for the isolated coil and metalate structures, the fragments of interest were manually extracted from geometry-optimized precipitate structures using ASE’s Graphical User Interface^77^ and centered in the unit cells. Precise modification of the cell vectors without affecting the molecular geometries was then achieved using a purpose-built interactive Python script interfaced with ASE (see Section S6). Visualizations of the simulation boxes were produced in VESTA 3.5.8.^82^ Relative uncorrected ligand energies and relative counterion corrections were obtained from differences in single-point energies for the relevant simulation boxes. The relative corrected ligand strain energies were calculated by summing the uncorrected energies and the counterion repulsion corrections. Ground-state electronic energies given in Table 3 used to calculate metalate displacement energies, Δ*U*_ex_, were the final SCF energies from geometry optimizations for the precipitate structures and single-point energies for the isolated metalate simulation boxes. For the latter, the electronic minimization method for the SCF calculation was changed to EDFT due to stability issues with the default density-mixing scheme.

### Hirshfeld Surface Analysis

Hirshfeld surfaces were generated, analyzed, and visualized using *CrystalExplorer*.^83^ The percentages of the surfaces occupied by fragment patches corresponding to different intermolecular contacts were obtained by summing the percentage surface areas occupied by the relevant atom–atom contacts.

### Noncovalent Interaction (NCI) Plots and Quantum Theory of Atoms in Molecules (QTAIM) Analysis

NCI plots and QTAIM graphs were generated using the CRITIC2 program.^84−87^ Valence-only input electron densities were loaded from *.cube files generated using the castep2cube utility. For NCI plot generation, the density cubes were core-augmented using CRITIC2’s internal core density grids and sampled between selected fragments from the crystal structure on a uniform grid with 0.15 Bohr spacing for *s* vs sign(λ)ρ plots, and 0.10 Bohr spacing for three-dimensional *s*-isosurface plots. For *s* vs sign(λ)ρ plots, each [H**L**]^+^ molecule was treated as a separate fragment so that the ligand···ligand interactions were captured. For *s*-isosurface plots, all [H**L**]^+^ molecules were combined into one fragment to exclude [H**L**]^+^···[H**L**]^+^ interactions from the visualization. In each case, the density cutoff value was 0.2 au. Graphical output was processed using VMD 1.9.3,^88^ and the *s* vs sign(λ)ρ data was plotted using Origin 2021b.^89^ For QTAIM analysis, no core augmentation was used to preserve the gradient of the modeled electron density. CRITIC2’s automatic CP localization algorithm was used to generate critical point reports and coordinate files. The atoms and critical points of interest were extracted from the coordinate files and visualized using VESTA 3.5.8.^82^

## Supporting Information Available

PXRD and DSC-PXRD patterns, further computational analysis, optimized atomic coordinates and energies, and purpose-built python script (PDF)
