# Supplementary material for: Selective Gold Precipitation by a Tertiary Diamide Driven by Thermodynamic Control
Source: Inorg Chem. 2024 May 9;63(20):9332–45. doi: 10.1021/acs.inorgchem.4c01279 (PMC11110006; doi:10.1021/acs.inorgchem.4c01279)
Supplement: Supplementary file 1 — ic4c01279_si_001.pdf [file ic4c01279_si_001.pdf]

Supplementary Information

Selective gold precipitation by a tertiary diamide driven by thermodynamic control

Susanna S. M. Vance,[1] Mateusz Mojsak,[1] Luke M. M. Kinsman,[1] Rebecca Rae,[1] Caroline Kirk,[1] Jason B. Love[1]* and Carole A. Morrison[1]*

[1]EaStCHEM School of Chemistry, University of Edinburgh, Edinburgh EH9 3FJ

* Carole.Morrison@ed.ac.uk *jason.love@ed.ac.uk

Section 1: X-ray crystal structure of [HL][$GaCl_4$] 2

Section 2: Output from Pawley refinements of PXRD datasets and DSC-PXRD datasets 3

Section 3: Computational modelling 13

Section 4: Hirshfeld surfaces and 2D NCI plots 14

Section 5: Optimised atomic coordinates and energies 17

Section 6: Python script for preparing model unit cells 27

Section 7: Crystallographic tables 32

References 33

Section 1: X-ray crystal structure of [HL][$GaCl_4$]

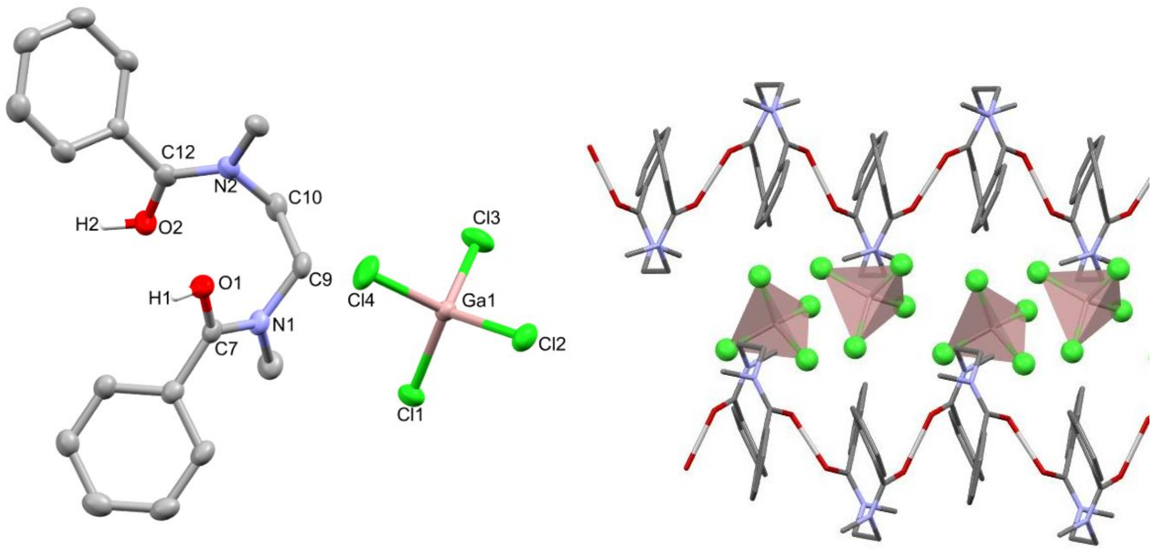

Figure S1. X-ray crystal structure of [HL][$GaCl_4$] – The x-ray structure obtained for [H**L**][$GaCl_4$] is very similar to that obtained for [H**L**][$FeCl_4$], with the metalates slotting into grooves between the ligand coils (right). Minimal variation was observed between the geometrical parameters for both structures (N1-C9-C10-N2 = 83.8(4)° and O1(H1)---O1a = 2.444(4) Å in [H**L**][$GaCl_4$] compared to N-C-C-N torsional angle = 88.2(8)° and O(H)---O = 2.445(8) Å in [H**L**][$FeCl_4$]). All hydrogen atoms except those involved in hydrogen bonding have been removed for clarity. Displacement ellipsoids drawn at 50% probability. Atom colors: grey = carbon, red = oxygen, blue = nitrogen, white = hydrogen, green = chlorine and pink = gallium.

Section 2: Output from Pawley refinements of PXRD datasets and DSC-PXRD datasets

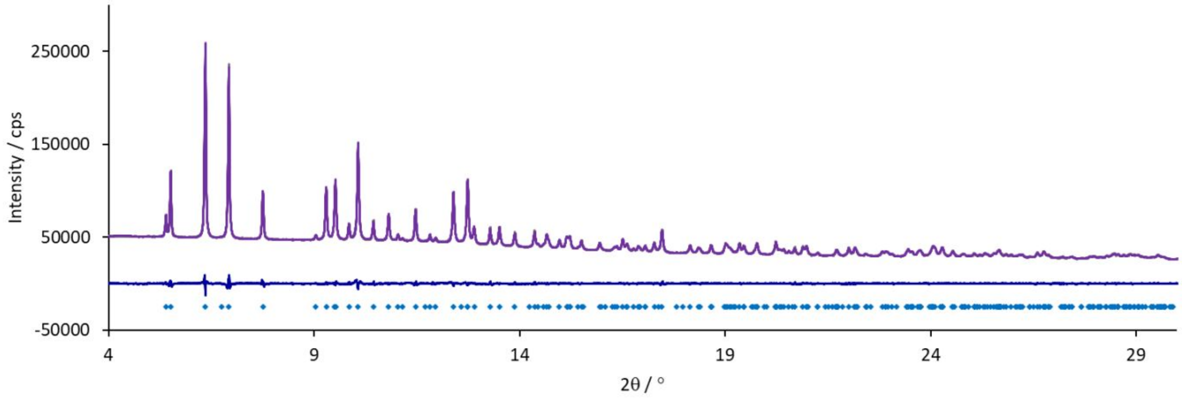

Unit Cell Parameter	Single Crystal	Pawley Fit
a	12.0848(7)	12.27990(9)
b	16.9574(2)	17.16410(15)
c	16.5644(10)	16.80361(10)
α	90	90
β	142.074(13)	142.6550(4)
γ	90	90

Figure S2. PXRD Pawley Refinement of [HL][$AuCl_4$] – The observed pattern of the bulk [H**L**][$AuCl_4$] precipitate, recorded on the high-resolution powder diffraction beamline (I11) at the Diamond Light Source, is shown in grey with the fit calculated using a Pawley refinement shown in purple. The difference profile is shown in dark blue and tick marks in light blue. An excellent match between the refined unit cell parameters from analysis of the PXRD data with the unit cell parameters from the single crystal XRD data refinement confirms the two phases are the same, with only slight variations in the unit cell parameters (see table above).

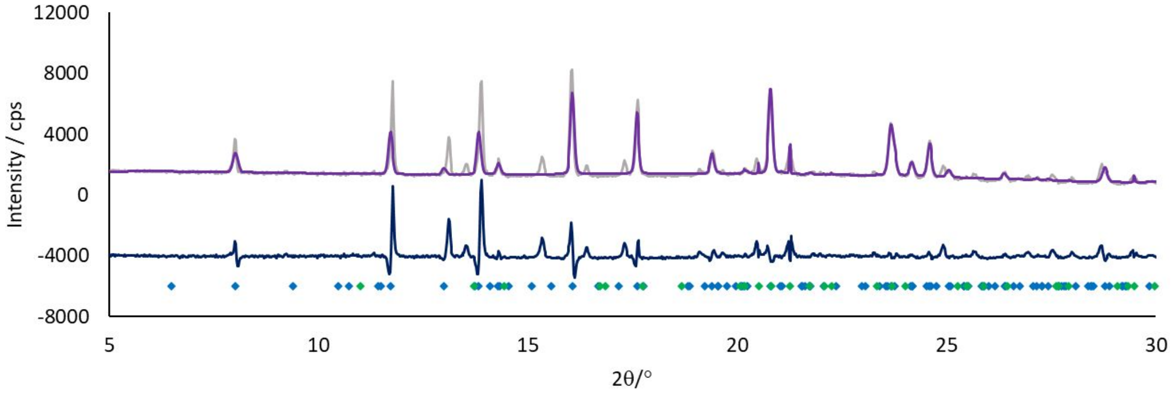

Unit Cell Parameter	Single Crystal	Pawley Fit
a	8.6732(2)	8.771(2)
b	18.8392(7)	18.813(6)
c	13.8641(5)	14.138(6)
α	90	90
β	104.665(3)	105.67(3)
γ	90	90

Figure S3. PXRD Pawley Refinement of [HL][$GaCl_4$] – The observed pattern of the bulk [H**L**][$GaCl_4$] precipitate is shown in grey with the fit calculated using a multiphase Pawley refinement shown in purple. The difference profile is shown in dark blue and tick marks associated with the [H**L**][$GaCl_4$] refined single crystal data are shown in light blue and those associated with the ligand (taken from the CCDC structure of **L** under collection code 1150349) are shown in green. Although the fit was poor, the refined unit cell parameters from analysis of the PXRD data and the unit cell parameters from the single crystal XRD data refinement are similar (see table above). Furthermore, the fit suggests the single crystal phase is the major phase present in the precipitate, with the unmatched peaks likely corresponding to another phase, such as a polymorph of the single crystal phase.

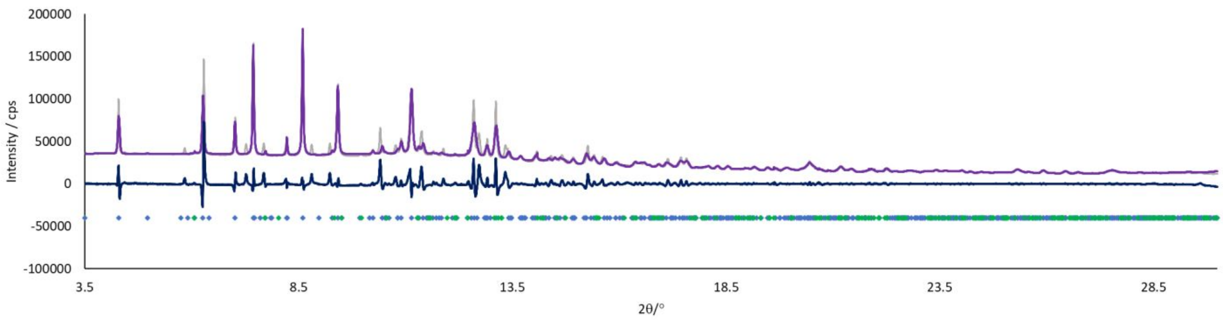

Unit Cell Parameter	Single Crystal	Pawley Fit
a	8.62820(10)	8.5181(5)
b	19.0047(3)	19.0664(19)
c	13.8701(3)	13.9777(12)
α	90	90
β	105.557(2)	104.932(10)
γ	90	90

Figure S4. PXRD Pawley Refinement of [HL][$FeCl_4$] – The observed pattern of the bulk [H**L**][$FeCl_4$] precipitate, recorded on the high-resolution powder diffraction beamline (I11) at the Diamond Light Source, is shown in grey with the fit calculated using a multiphase Pawley refinement shown in purple. The difference profile is shown in dark blue and tick marks associated with the [H**L**][$FeCl_4$] refined single crystal data are shown in light blue and those associated with the ligand (taken from the CCDC structure of **L** under collection code 1150349) are shown in green. Although the fit was poor, the refined unit cell parameters from analysis of the PXRD data and the unit cell parameters from the single crystal XRD data refinement are similar (see table above). Furthermore, the fit suggests the single crystal phase is the major phase present in the precipitate, with the unmatched peaks likely corresponding to another phase, such as a polymorph of the single crystal phase.

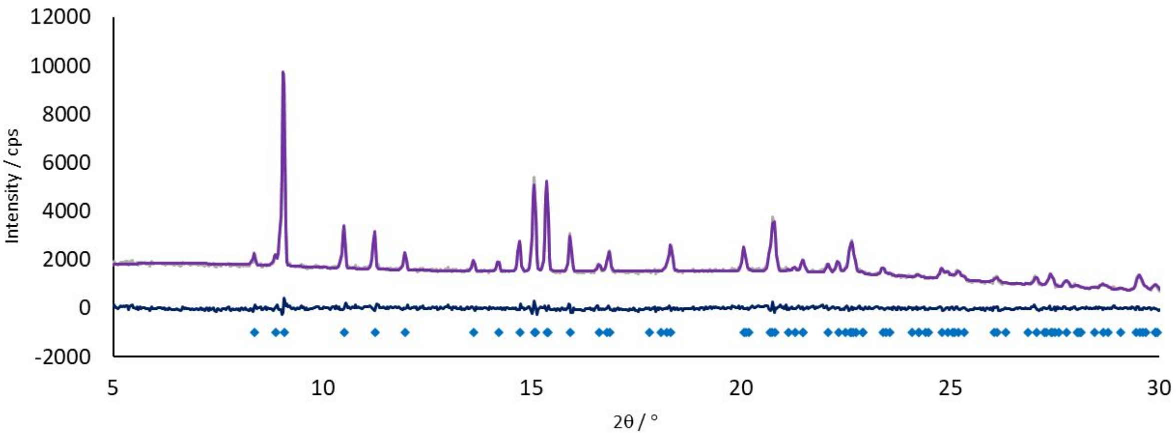

Unit Cell Parameter	Single Crystal	Pawley Fit
a	8.7720(4)	8.9032(8)
b	11.1752(5)	11.2124(8)
c	12.0751(6)	12.1020(13)
α	113.632(4)	113.730(5)
β	103.282(4)	103.176(10)
γ	97.177(9)	97.387(11)

Figure S5. PXRD Pawley Refinement of [HL]$_2$[SnCl$_6$](H$_2$O)$_2$ – The observed pattern of the bulk [H**L**]$_2$[SnCl$_6$](H$_2$O)$_2$ precipitate is shown in grey with the fit calculated using a Pawley refinement shown in purple. The difference profile is shown in dark blue and tick marks in light blue. An excellent match between the refined unit cell parameters from analysis of the PXRD data with the unit cell parameters from the single crystal XRD data refinement confirms the two phases are the same, with only slight variations in the unit cell parameters (see table above).

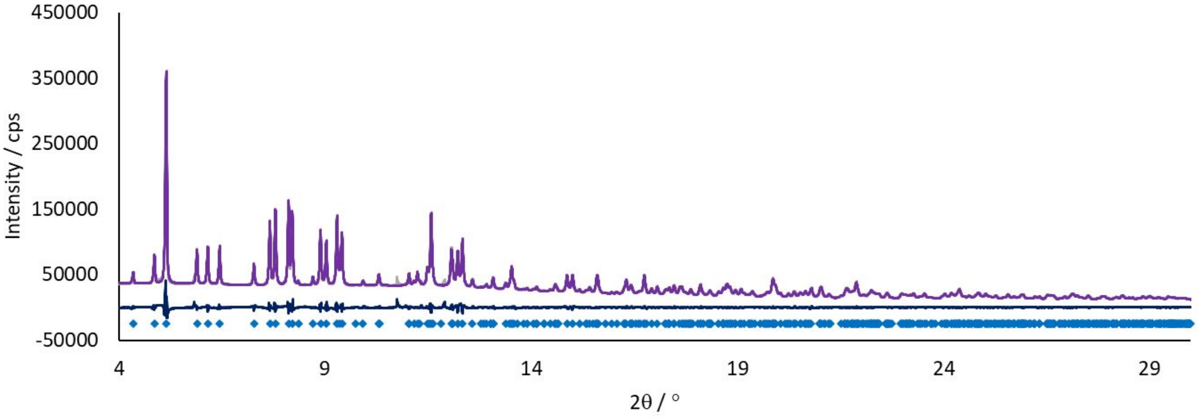

Unit Cell Parameter	Single Crystal	Pawley Fit
a	8.50150(10)	8.65925(9)
b	10.9005(2)	11.02055(16)
c	11.7800(2)	11.84195(17)
α	108.290(2)	108.958(1)
β	97.0390(10)	96.716(1)
γ	106.770(2)	107.326(1)

Figure S6. PXRD Pawley Refinement of [HL]$_2$[SnCl$_6$] – The observed pattern of the bulk [H**L**]$_2$[SnCl$_6$] precipitate, recorded on the high-resolution powder diffraction beamline (I11) at the Diamond Light Source, is shown in grey with the fit calculated using a Pawley refinement shown in purple. The difference profile is shown in dark blue. An excellent match between the refined unit cell parameters from analysis of the PXRD data with the unit cell parameters from the single crystal XRD data refinement confirms the two phases are the same, with only slight variations in the unit cell parameters (see table above). The remaining unmatched peaks correspond to residual ligand in the starting material.

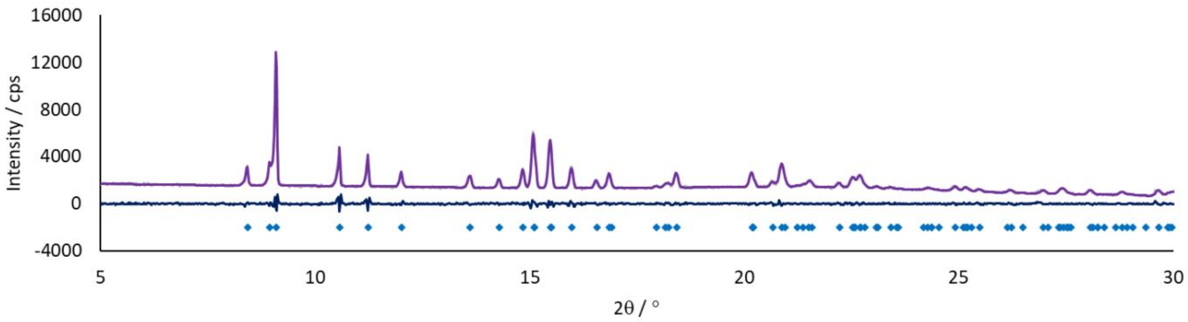

Unit Cell Parameter	Single Crystal	Pawley Fit
a	8.7579(10)	8.8914(4)
b	11.0954(14)	11.1667(6)
c	12.0668(15)	12.0992(9)
α	113.978(5)	114.058(4)
β	103.941(5)	103.711(7)
γ	96.913(5)	97.211(7)

Figure S7. PXRD Pawley Refinement of [HL]$_2$[PtCl$_6$](H$_2$O)$_2$ – The observed pattern of the bulk [H**L**]$_2$[PtCl$_6$](H$_2$O)$_2$ precipitate is shown in grey with the fit calculated using a Pawley refinement shown in purple. The difference profile is shown in dark blue and tick marks in light blue. An excellent match between the refined unit cell parameters from analysis of the PXRD data with the unit cell parameters from the single crystal XRD data refinement confirms the two phases are the same, with only slight variations in the unit cell parameters (see table above).

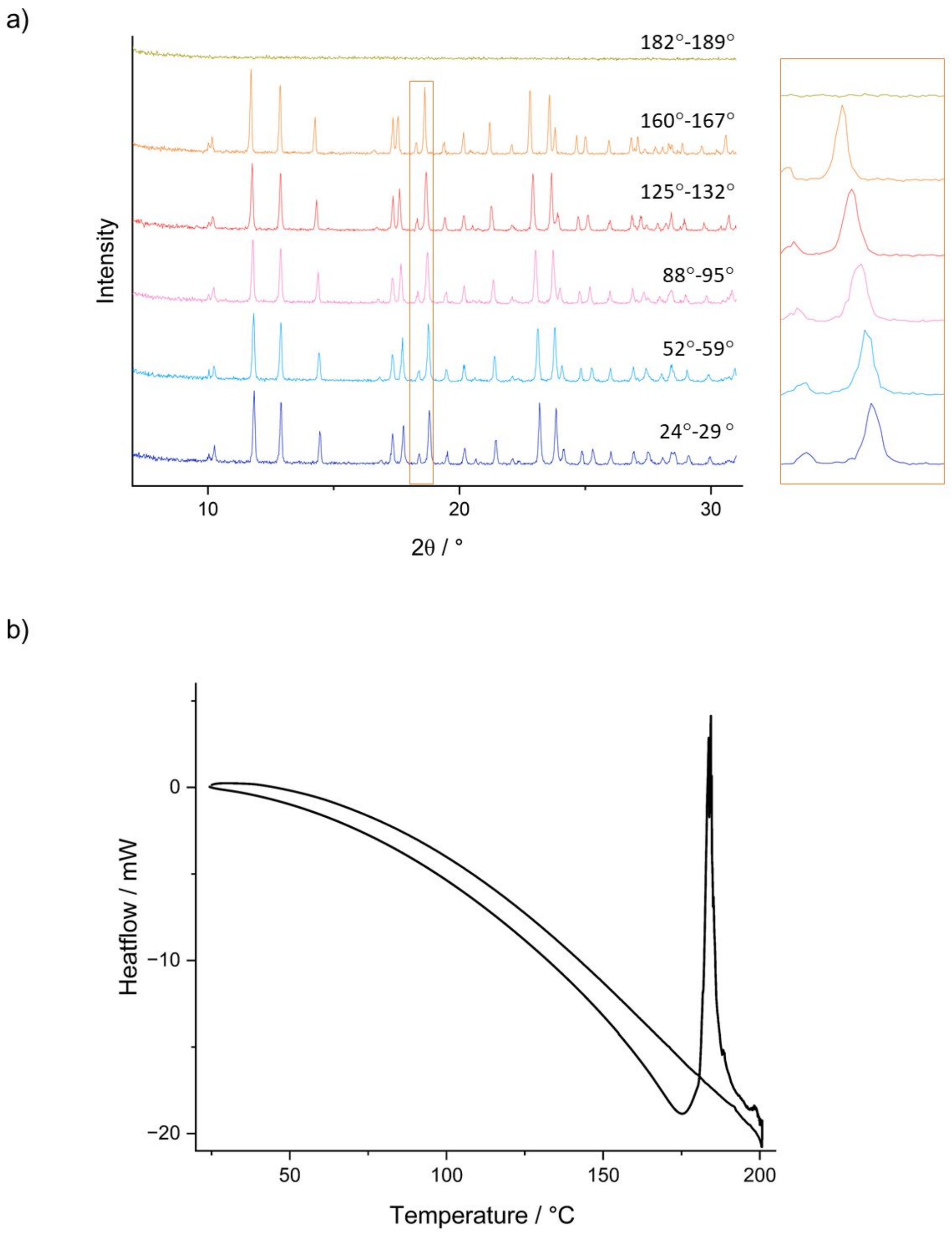

UC Parameter	24 °C – 29 °C	52 °C – 59 °C	88 °C – 95 °C	125 °C – 132 °C	182 °C – 189 °C
a	12.2946(8)	12.3298(7)	12.3757(7)	12.4290(7)	12.4905(6)
b	17.1857(15)	17.2159(14)	17.2565(15)	17.3019(14)	17.3539(11)
c	16.8214(10)	16.8689(9)	16.9243(9)	16.9885(8)	17.0657(7)
β	142.640(3)	142.749(3)	142.903(3)	143.086(3)	143.268(2)

Figure S8. ***In situ*** **DSC – PXRD measurements of [HL][$AuCl_4$] – a)** Selected PXRD patterns from the 20 – 200 °C heat cycle, showing the slight peak shift of observed diffraction peaks to lower scattering angles (orange selection, right of figure (a)) and eventual loss of crystallinity at *ca.* 180 °C for the [H**L**][$AuCl_4$] precipitate. Pawley refinement of the unit cell parameters for the selected patterns above

confirms thermal expansion with an increase in all unit cell parameters noted (see above table). **b)** DSC curve over the 20 – 200 °C heat cycle, showing a large endothermic peak at *ca.* 180 °C corresponding to decomposition of the precipitate.

a)

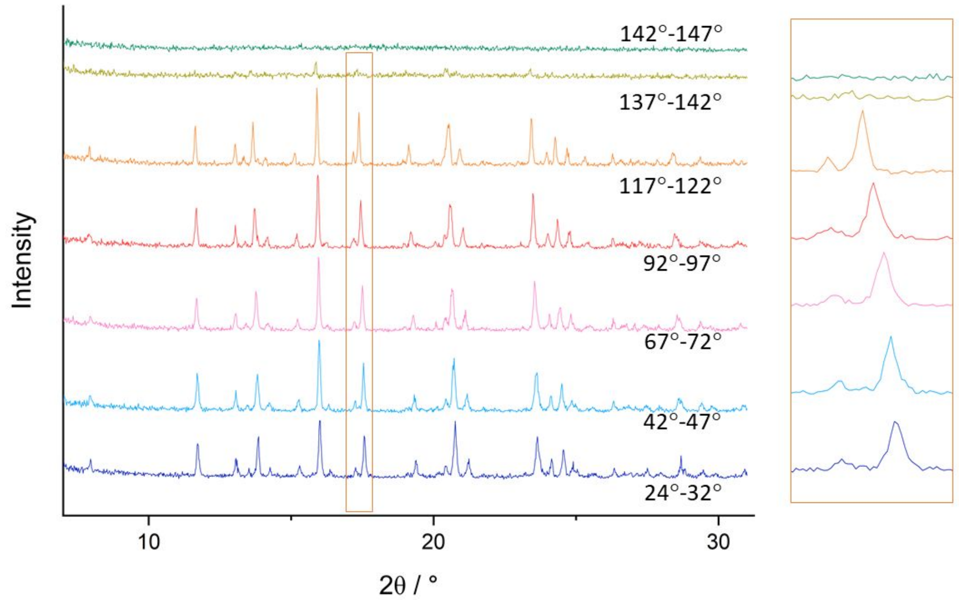

b)

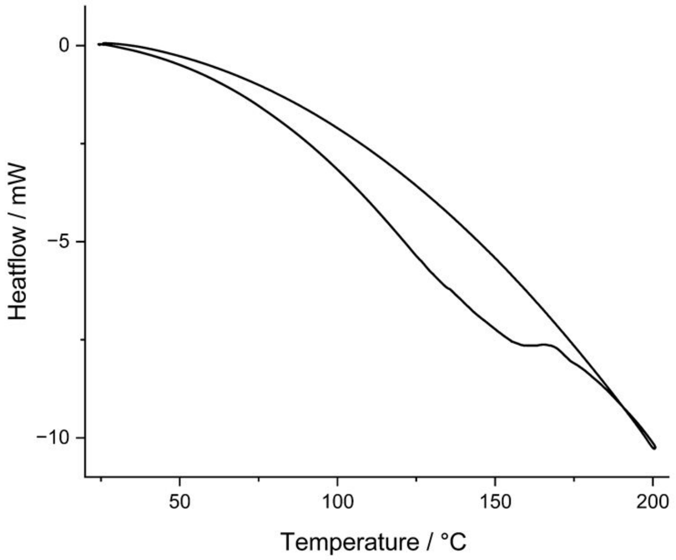

Figure S9. ***In situ*** **DSC – PXRD measurements of [HL][$GaCl_4$] – a)** Selected PXRD patterns from the 20 – 200 °C heat cycle, showing thermal expansion of the crystallites as diffraction peaks shift to lower scattering angles with an eventual loss of crystallinity at *ca.* 142 °C. Pawley refinement of the unit cell parameters to further confirm thermal expansion was not possible with this data set due to the high signal to noise ratio which results from the short collection time implemented in these measurements. **b)** DSC curve over the 20 – 200 °C heat cycle, showing a diffuse peak at an onset temperature of *ca.* 130 °C and ending at *ca.* 150 °C, which corresponds to a loss of crystallinity in the PXRD pattern.

a)

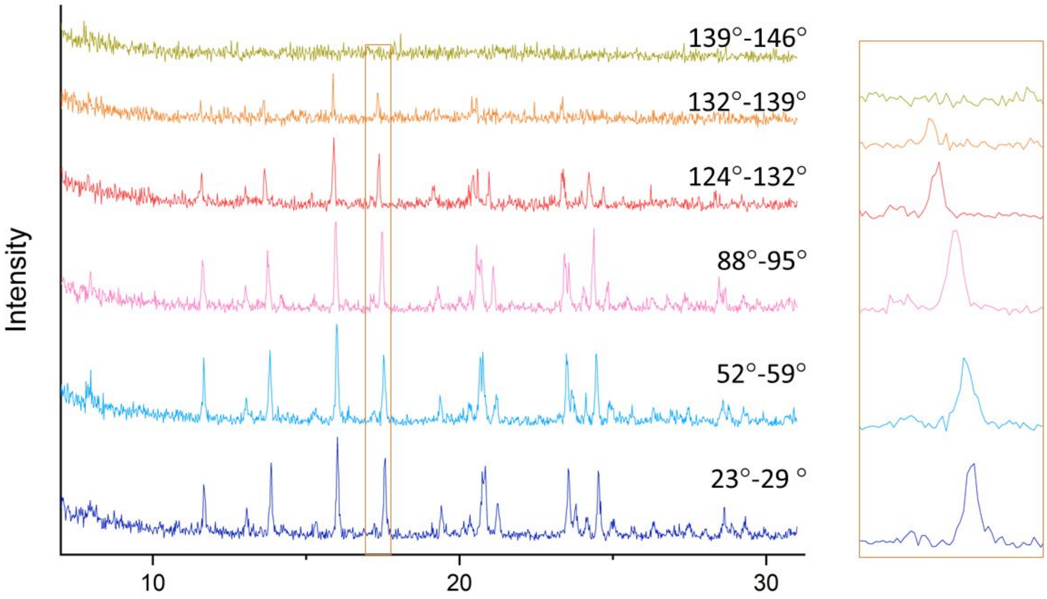

b)

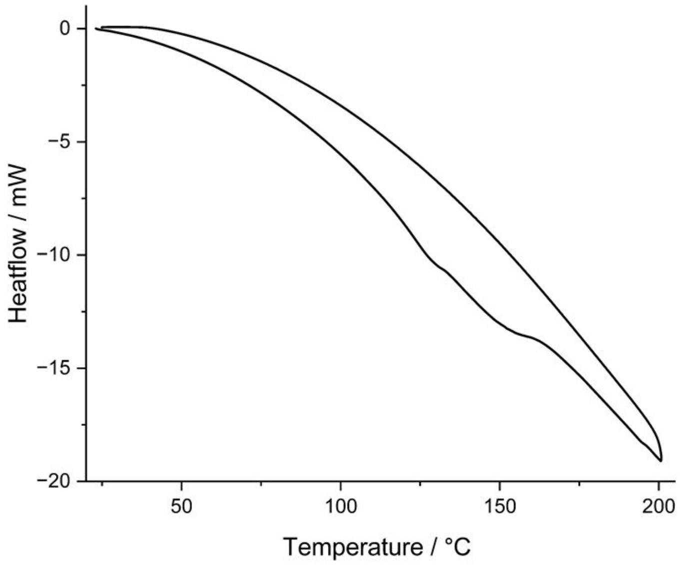

Figure S10. *In situ* DSC – PXRD measurements of [HL][$FeCl_4$] – a) Selected PXRD patterns from the 20 – 200 °C heat cycle, showing the slight peak shift of observed diffraction peaks to lower scattering angles with an eventual loss of crystallinity at *ca.* 139 °C. Again, refinement of the unit cell parameters was not possible with this data set due to very high signal to noise, arising from the presence of iron in the sample and the short collection time of each pattern. **b)** DSC curve over the 20 – 200 °C heat cycle, showing a diffuse peak at an onset temperature of *ca.* 120 °C and ending at *ca.* 160 °C, which corresponds to a loss of crystallinity in the PXRD pattern.

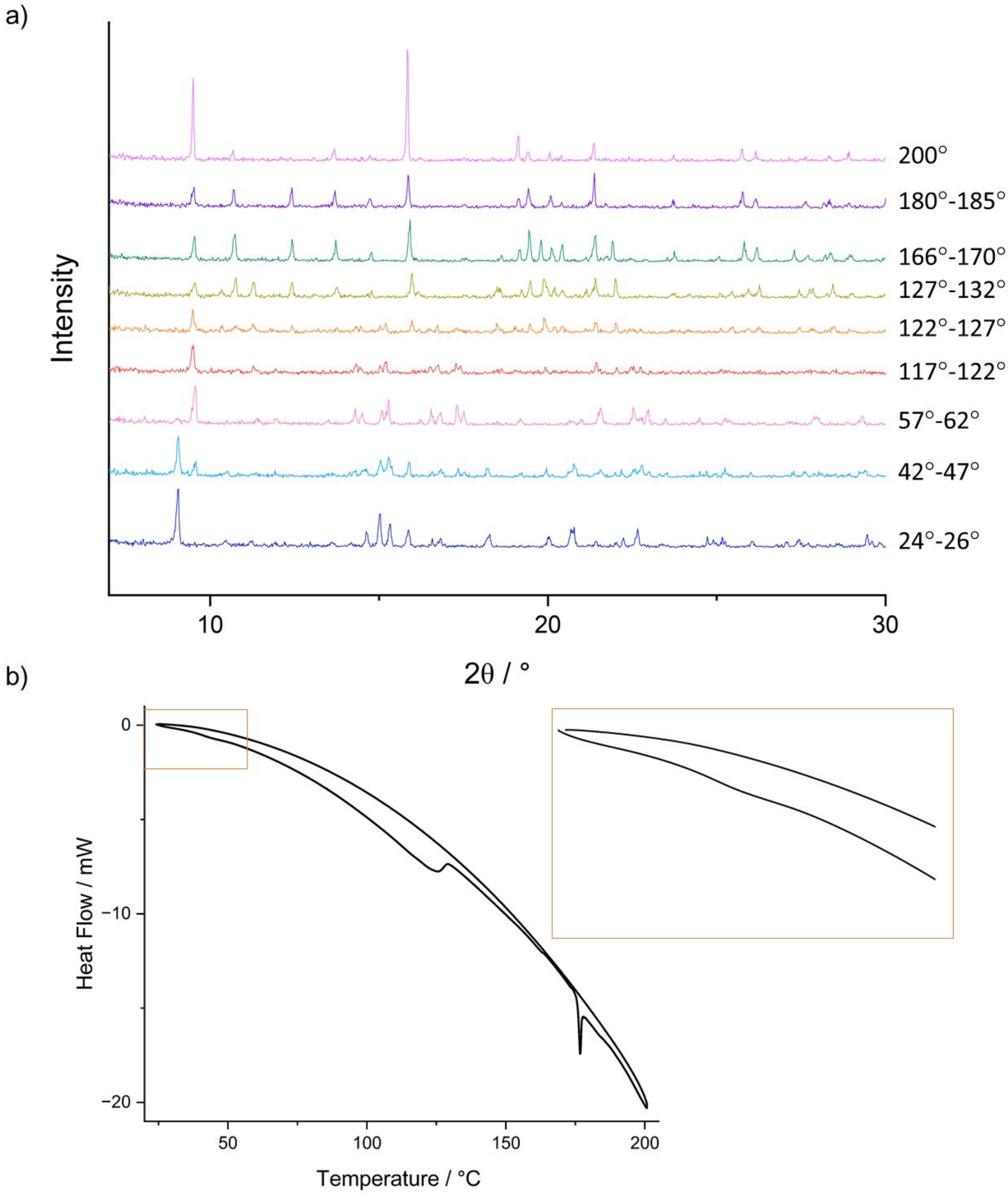

UC Parameter	Hydrated (SC-XRD)	24 °C – 26 °C	Dehydrated (SC-XRD)	57 °C – 62 °C
a	8.7720(4)	8.8988	8.50150(10)	8.7133
b	11.1752(5)	11.2463	10.9005(2)	11.0730
c	12.0751(6)	12.1008	11.7800(2)	11.8658
α	113.632(4)	113.655	108.290(2)	109.128
β	103.282(4)	103.005	97.0390(10)	96.593
γ	97.177(4)	97.447	106.770(2)	107.574
Cell Volume	1023.69(9)	1047.517	964.78(3)	1001.703
GoF	n/a	13.27	n/a	14.77

Figure S11. ***In situ*** **DSC – PXRD measurements of $[H\mathbf{L}]_2[SnCl_6](H_2O)_2$ – a)** Selected PXRD patterns from the 20 – 200 °C heat cycle for the $[H\mathbf{L}]_2[SnCl_6](H_2O)_2$ precipitate, detailing three transitions. The first transition corresponds to a loss of channel water and occurs at an onset temperature of *ca.* 42 °C with complete water loss observed at 57 °C, shown by a shift in the peaks to higher scattering angles without an overall change in the powder pattern. Although Pawley refinement of this data was not possible due to poor signal to noise, the data were indexed, with refined unit cell parameters similar to those determined by analysis of single crystal data for both phases (see table above). The unit cell parameters of the dehydrated phase show a decrease in all cell lengths and the α and β angles, resulting in a reduction in unit cell volume from the loss of water at 57 °C – 62 °C. The second transition occurs at *ca.* 127 °C, which appears to be a second order transition such as a glass transition, with a change in the PXRD pattern observed. The final transition occurs at 175 °C and corresponds to the endotherm melting point of the ligand, which has a literature melting point of 177 °C.[1] The remaining crystalline phase must therefore correspond to a $SnCl_x$ species, which explains the good scattering and apparent high crystallinity of this phase. **b)** DSC curve over the 20 – 200 °C heat cycle, showing a small bowing endotherm peak at *ca.* 45 °C which correlates with the loss of channel water noted in the PXRD pattern (see orange insert). A further monotropic phase transition is observed at *ca.* 125 °C with the final transition at 175 °C corresponding to the melting point endotherm of the ligand.

a)

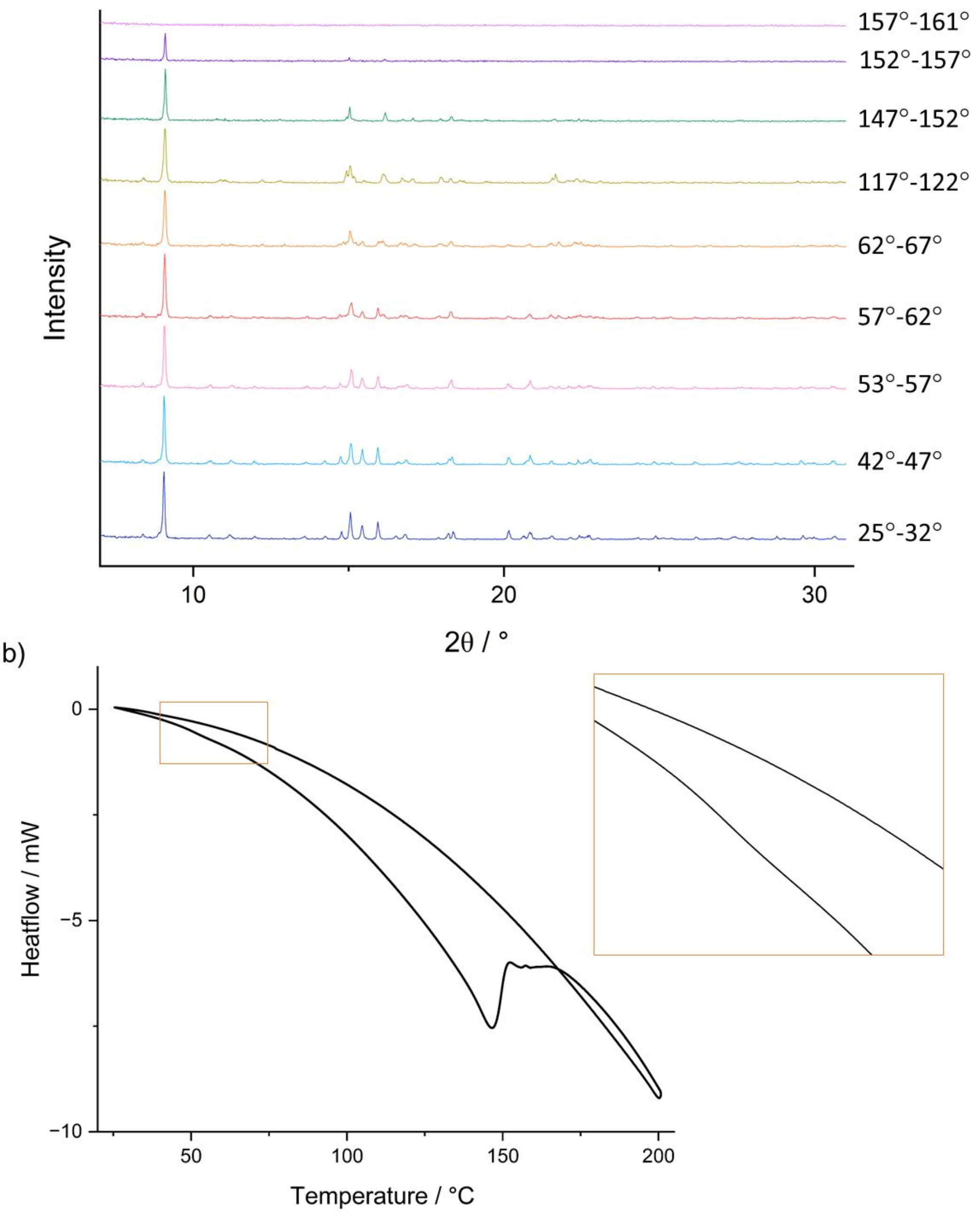

Figure S12. ***In situ*** **DSC – PXRD measurements of $[HL]_2[PtCl_6](H_2O)_2$ – a)** Selected PXRD patterns from the 20 – 200 °C heat cycle for the $[H\mathbf{L}]_2[PtCl_6](H_2O)_2$ precipitate, showing two transitions. The first transition corresponds to a loss of lattice water and occurs at an onset temperature of *ca.* 62 °C with a gradual loss of crystallinity observed from this temperature, confirming that water of crystallisation is important in crystal formation for this precipitate. The second transition occurs at *ca.* 150 °C, whereby the sample melts, losing all crystallinity, with an amorphous solid formed upon cooling. **b)** DSC curve over the 20 – 200 °C heat cycle, showing a small bowing endotherm peak at *ca.* 60 °C which correlates with the loss of lattice water in the PXRD pattern. A further transition occurs at *ca.* 150 °C and corresponds to the melting endotherm.

Section 3: Computational modelling

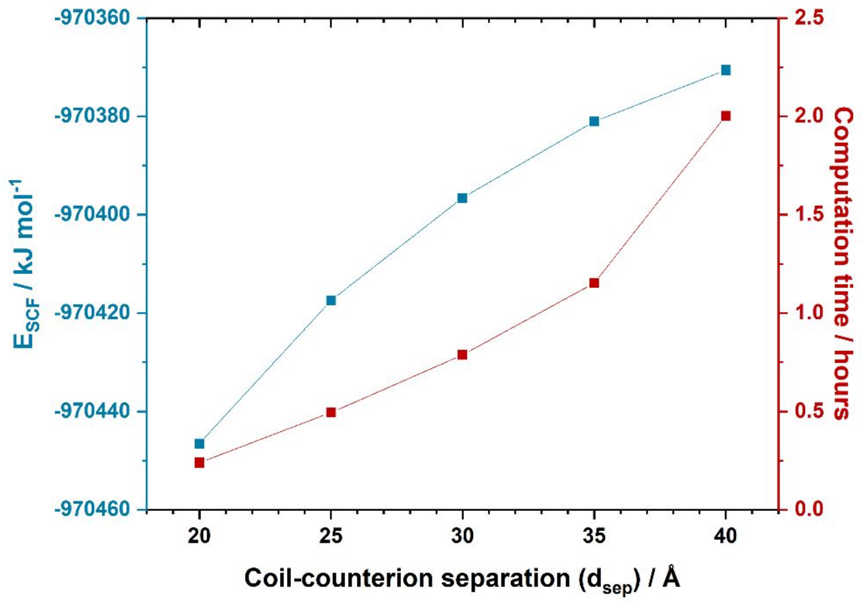

Figure S13. Determination of Optimum Simulation Box size for the Extracted Ligand Coils – The optimum width of the vacuum layer in the simulation boxes was determined through a series of single point energy calculations, whereby the box size was incrementally increased by changing the d_{sep} values. At a d_{sep} value of 35 Å, the predicted change in energy when the vacuum layer width was increased by 1 Å was 2.5 kJ mol^{-1} Å^{-1}. With the maximum difference in the extension of the coil from its central axis 0.29 Å across all structures, a d_{sep} value of 35 Å was deemed appropriate to sufficiently capture energy differences greater than approximately 1 kJ mol^{-1}.

Table S1. Calculation of ΔU_{ex} – The energy required to exchange each metalate with $[AuCl_4]^-$ in the precipitate was calculated according to equations 2 and 3 in the main text using the ground state electronic energies given below, with the exchange energy given in the final column.

M	Ground State Electronic Energy / kJ mol^{-1}			ΔU_{ex} / kJ mol^{-1}
	**$[HL][MCl_4]$**	**$[Na][MCl_4]$**		
**Au**	−1652511.24	−1971354.75		0.00
**Ga**	−496914.39	−815738.96		18.93
**Fe**	−372739.85	−691549.27		34.09
	**$[HL]_2[MCl_6]\cdot 2H_2O$**	**$[Na]_2[MCl_6]$**	**H_2O**	
**Sn**	−695985.90	−1424782.38	−45606.98	104.51
**Pt**	−1756190.326	−2484970.53		120.78

Table S2. Calculation of ΔG_{ex} for $[HL][GaCl_4]$ – ΔU_{ex} was used in conjunction with the zero-point energy (ZPE) and entropy (S) corrections given below to calculate ΔG_{ex} for the gallium complex, given the smallest exchange energy was observed for this structure. ΔG_{ex} was calculated as follows:

$$\Delta G_{ex} = \Delta ZPE + \Delta U_{ex} - T\Delta S \quad (1)$$

Where T=293 K and ΔZPE and ΔS are the change in ZPE and S respectively. With very small variation between complexes the ZPE had a negligible effect on the final energy, with the major contribution to the final Gibbs free energy due to the entropic contribution.

Species	Zero Point Energy / kJ mol^{-1}	Entropy / kJ mol^{-1} K^{-1}	ΔG_{ex} / kJ mol^{-1}
[H**L**][$AuCl_4$]	934.60	54.03	314.86
[H**L**][$GaCl_4$]	934.74	55.04	
$[AuCl_4]^-$	12.33	0.39	
$[GaCl_4]^-$	12.64	0.38	

Section 4: Hirshfeld surfaces and 2D NCI plots

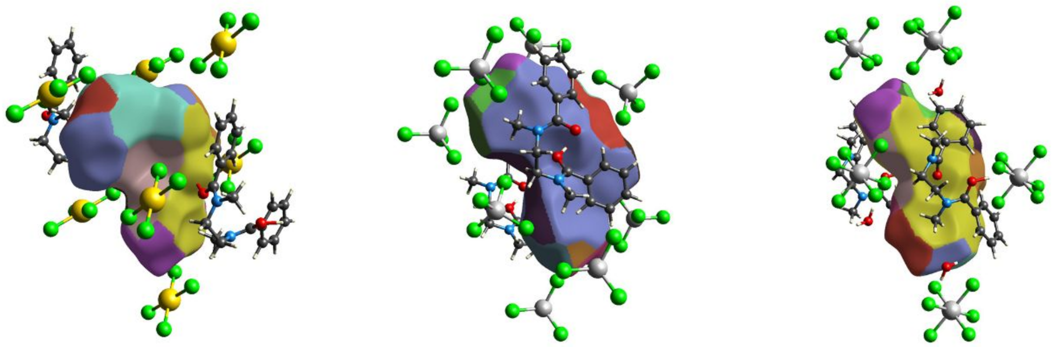

a) [H**L**][$AuCl_4$]   b) [H**L**][$GaCl_4$]   c) $[H\mathbf{L}]_2[SnCl_6](H_2O)_2$

Figure S14. Hirshfeld surface fragment patches – Fragment patches on Hirshfeld surfaces for one $[H\mathbf{L}]^+$ unit for three representative structures, namely **a)** gold **b)** gallium and **c)** tin. Different colours on the plots represent areas of close contacts with neighbouring molecules. Analysing these colour patches reveals that the ligand in the gold structures uses 3.8% of its surface area to bind to the gold metalate, 3.4% for gallium, iron and tin and 3.2% for platinum. This consolidates the conclusions drawn in this study that the gold metalate is the most efficiently packed in the crystal lattice and therefore maximises intermolecular interactions with the ligand.

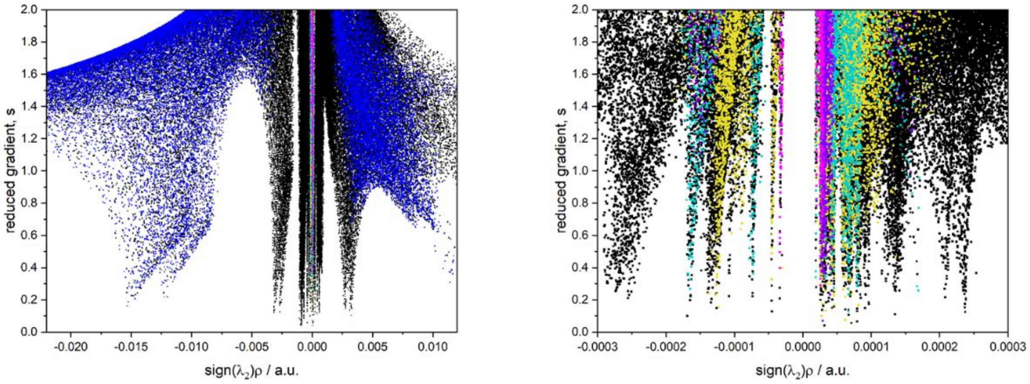

a) [H**L**][$AuCl_4$]

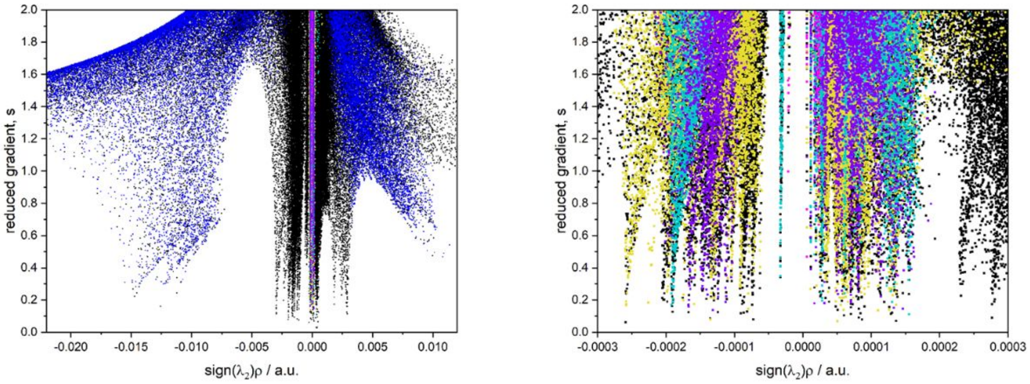

b) [H**L**][$GaCl_4$]

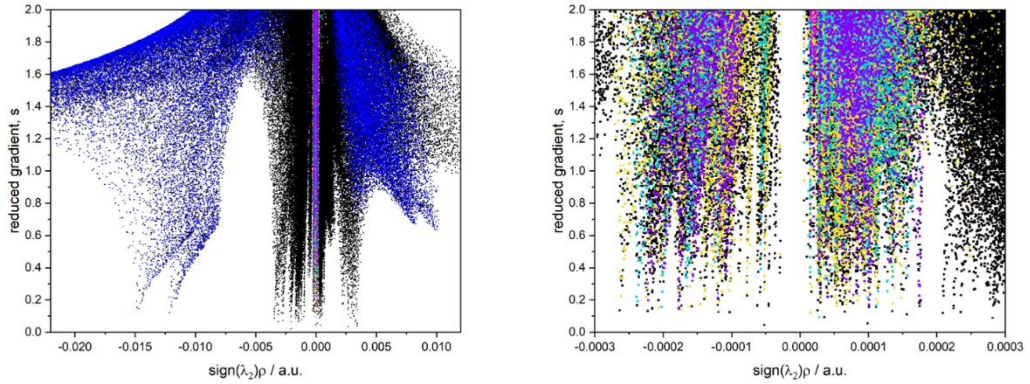

c) [H**L**][$FeCl_4$]

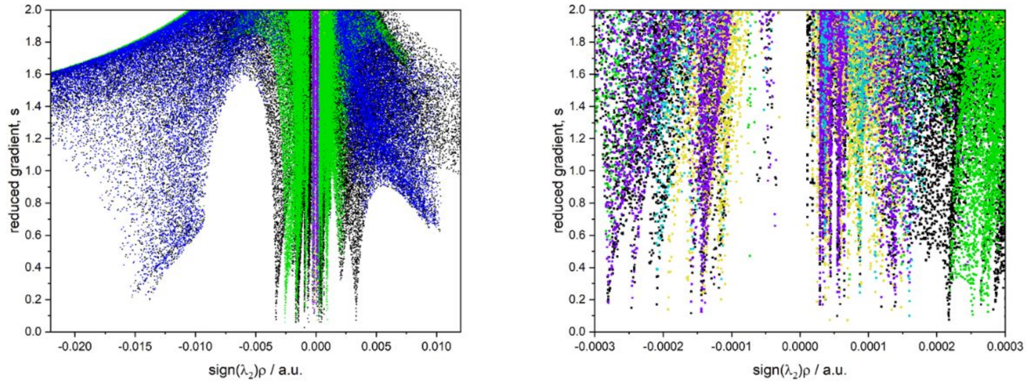

d) [H**L**]$_2$[$SnCl_6$](H_2O)$_2$

[H**L**]$^+$··· [H**L**]$^+$ CH_2···MCl_x MCl_x···MCl_x CH_3···MCl_x H Bond

H_2O···MCl_x ···LH Ar···MCl_x

Figure S15. 2D NCI plot analysis – 2D Reduced gradient, s vs sign(λ)ρ plots for the **a)** [H**L**][$AuCl_4$] **b)** [H**L**][$GaCl_4$], **c)** [H**L**][$FeCl_4$] and **d)** [H**L**]$_2$[$SnCl_6$](H_2O)$_2$ crystal structures, coloured to highlight the nature of the interactions according to the legend, with the full reduced density plot shown on the left and an excerpt of the plot shown on the right to highlight the dispersive interactions. Unassigned interactions (shown in black) correspond to the remaining [H**L**]$^+$···[H**L**]$^+$ interactions. Features approaching zero on the Y-axis indicate a stationary point on the reduced density surface (i.e., a non-covalent interaction), and the value of the intercept on the X-axis indicates whether the interaction is attractive (sign(λ_2)ρ < 0), repulsive (sign(λ_2)ρ > 0) or dispersive (sign(λ_2)ρ ≈ 0) in nature.

Analysis of these plots indicates that the strong intermolecular interactions (O-H···O hydrogen bonds (blue) and [H**L**]$^+$···[H**L**]$^+$ interactions (black)) are ligand···ligand based and broadly similar in all structures, excluding the [MCl_6]$^{2-}$···H_2O interaction only present in the [$SnCl_6$]$^{2-}$ plot (green troughs (d)). Therefore, in order to identify variations in the intermolecular interactions between structures, detailed analysis of the plots must focus on the dispersion interactions close to sign(λ_2)ρ = 0 which characterise the [H**L**]$^+$···[MCl_x]$^{y-}$ and [MCl_x]$^{y-}$···[MCl_x]$^{y-}$ interactions (Figure S15, right). The most notable difference in this area of the plots is that the host···guest interactions seem to increase in strength in the reverse order of selectivity, with the weakest interactions (closest to sign(λ_2)ρ = 0) observed in the [H**L**][$AuCl_4$] plot, followed by [H**L**][$GaCl_4$] and [H**L**][$FeCl_4$], while the strongest interactions are observed in the [H**L**]$_2$[$SnCl_6$](H_2O)$_2$ plot. However, this analysis does not take the multiplicity of the individual interactions into account, and therefore this observation is not indicative of overall host···guest interaction strengths. The 2D plot for [H**L**][$AuCl_4$] depicts the CH_2···Au and Cl···Cl interactions as stabilising, consolidating their importance in precipitate stability, and although CH_2···metalate interactions appear more stabilising in the [H**L**][$GaCl_4$], [H**L**][$FeCl_4$] and [H**L**]$_2$[$SnCl_6$](H_2O)$_2$ plots, this is due to a change in the type of interaction. The CH_2 moiety interacts directly with the metal centre in the gold structure providing complete encapsulation of the metalate, whereas in the other structures the group interacts with the metalate through non-classical hydrogen bonds, which we would expect to be a stronger interaction but less significant in encapsulating the metalate. [H**L**]$^+$···metalate interactions appear at similar negative sign(λ_2)ρ values in the [H**L**][$GaCl_4$] and [H**L**][$FeCl_4$] plots however are more delocalised and shifted to a higher sign(λ_2)ρ in the iron structure, indicating a more destabilising character. Furthermore, a trace signature of the favourable [MCl_x]$^{y-}$···[MCl_x]$^{y-}$ interactions is observed in the [H**L**][$GaCl_4$] plot, while only present as a destabilising interaction in the [H**L**][$FeCl_4$] plot. Finally, fewer [H**L**]$^+$···[MCl_x]$^{y-}$ interactions are present in the 2D [H**L**]$_2$[$SnCl_6$](H_2O)$_2$ plot, with the [MCl_x]$^{y-}$···[MCl_x]$^{y-}$ interaction being completely absent.

Section 5: Optimised atomic coordinates and energies

Space group, space group number, unit cell parameters (not optimised) and optimised symmetry inequivalent coordinates for the geometry optimised complexes presented in this work are given below.

[HL][$AuCl_4$]

Space group name: C2/c
Symmetry Int. Tables number: 15

Unit cell parameters (not optimised):
a = 12.084800000000000
b = 16.957400000000000
c = 16.564400241174202
α = 12.084800000000000
β = 16.957400000000000
γ = 16.564400241174202

Optimised symmetry-inequivalent fractional atomic coordinates:

H	0.590963395000488	0.613136053844256	0.704218796758304
H	0.603685316580980	0.533066947620703	0.779732394002887
H	0.616935920470579	0.631968124028967	0.825303829425865
H	0.343019373499327	0.717111473471872	0.615954759301316
H	0.134706087962853	0.668065687528273	0.511189602145943
H	0.189382218932681	0.382391030060350	0.547526916840365
H	0.303436363492814	0.269542526038301	0.691505883417901
H	0.512314392770472	0.292678675422471	0.928783652831480
H	0.614946451087614	0.426956587451017	0.019898120977981
H	0.503946346318714	0.541307938229313	0.875445428667648
H	0.000000000000000	0.500000000000000	0.500000000000000
C	0.260372256303575	0.535807222546083	0.612635314821335
C	0.554664800914468	0.593400773920342	0.744146721713069
C	0.264074421428877	0.664727069195766	0.554049267088171
C	0.342691183987785	0.468723060229038	0.703411330093204
C	0.284443681040380	0.392214699732996	0.651306767881543
C	0.348492647862481	0.328707399920097	0.732956439426640
C	0.466924736288376	0.341544786632017	0.865190554583923
C	0.524370358949358	0.417793931988052	0.916989225130872
C	0.463844086609097	0.481441587852488	0.836784450562621
N	0.358488718680959	0.595061718019041	0.639152674454956
O	0.089286820736213	0.538485807225827	0.505211328626551
Cl	0.954444906855355	0.791887551567160	0.574038386006078
Cl	0.151138453062487	0.626109716846767	0.730434120779388
Au	0.250000000000000	0.750000000000000	0.75000000000000

LBFGS Final Enthalpy = -8.17260430E+004 eV

[HL][FeCl$_4$]

Space group name: P2_1
Symmetry Int. Tables number: 4

Unit cell parameters (not optimised):
a = 8.628200000000000
b = 19.004700000000000
c = 13.870100388842650
α = 90.000000000000000
β = 105.557000339573477
γ = 90.000000000000000

Optimised symmetry-inequivalent fractional atomic coordinates:

H	0.323668056846106	0.406923037988925	0.552857961539153
H	0.381114499369639	0.489195881057464	0.695223114254876
H	0.520556748497829	0.603027669921813	0.684648050119642
H	0.592691485049380	0.634927657656534	0.528708024305099
H	0.529502279290104	0.553578568558478	0.383996944014711
H	0.500830165941605	0.301095673678471	0.517637404800813
H	0.620033217165901	0.378744421953643	0.522219616356034
H	0.656117835727497	0.304080930763871	0.454769716516888
H	0.293940508769603	0.323844397735310	0.250716868703176
H	0.404958545136950	0.258681119599602	0.331405011760505
H	0.660815639602066	0.318673166463304	0.290576980155092
H	0.540386846818482	0.257348539129071	0.207541763393915
H	0.447994207034499	0.297209329784115	0.029580700812482
H	0.354597067626799	0.381881344638049	0.016753116243212
H	0.289393199042392	0.315323932855950	0.086987930829209
H	0.644605416868031	0.395177114944811	0.994935105558393
H	0.634104053607396	0.478103131084445	0.856014314870346
H	0.546318950136504	0.602150511837068	0.868639538131872
H	0.477918815100101	0.643144900989519	0.022486006053866
H	0.492913595765271	0.560658513671563	0.162543632858579
H	0.988189066504187	0.423686990890950	0.355090209380322
H	0.018522051115641	0.333006642301276	0.485495382134338
H	0.986515944018342	0.364395596472023	0.653740391417164
H	0.921561580261932	0.487824580867626	0.689457195799323
H	0.892825951597741	0.578995878557685	0.559046937857147
H	0.106870758074715	0.679828204109400	0.516001110566714
H	0.230864431716642	0.651165811571327	0.438282501372482
H	0.163692845934875	0.589726709731683	0.513944483965559
H	0.942085792487690	0.730200477997190	0.357287858062082
H	0.818618293678930	0.672875561566333	0.268835091899051
H	0.042228344034938	0.744906170388742	0.216805966569214
H	0.182768700618749	0.685911324745259	0.291063672072208
H	0.960423651647951	0.708345683178173	0.035415733947593
H	0.804997210750440	0.689442506298205	0.094859347939855
H	0.865782686982081	0.623917650647639	0.021005928429726
H	0.992317505734884	0.445913191540724	0.155664588038870
H	0.962135685391983	0.365718119395709	0.011554933734991

H	0.036275600845055	0.406403105802239	0.859323418289801
H	0.137988559079256	0.528771346457837	0.851435009802839
H	0.160064386745675	0.609833523981303	0.993118934968848
H	0.243407996690915	0.506796677552493	0.269382702413435
H	0.731908449847230	0.493425722805662	0.269887754839047
C	0.382237715101087	0.456865557557877	0.547099693094876
C	0.415169138978188	0.503332938575308	0.627605687230509
C	0.492344101743755	0.567039657379321	0.621313414420335
C	0.532904968526535	0.585112174162575	0.533718056542441
C	0.497590762341483	0.539390205194877	0.452285611041369
C	0.423616641737452	0.474681489104986	0.459064957506351
C	0.390900666808725	0.426303366531418	0.371781695134330
C	0.562295255032297	0.334984754302516	0.475329015843020
C	0.412737113228208	0.311089591902093	0.299918724577600
C	0.540161504320413	0.309602848181267	0.241293295403655
C	0.394287394651840	0.337570991850355	0.067224706111400
C	0.578410105980562	0.423951187207984	0.171714664510425
C	0.566281274521869	0.472156601101206	0.086561535255716
C	0.605887364498133	0.449234377116395	0.000091014575437
C	0.600467275278684	0.496117410592215	0.922214525421430
C	0.553061749245719	0.565600448314946	0.930072608873395
C	0.515086314515812	0.588850626508767	0.016483571452150
C	0.522663903278502	0.542483331749599	0.095178956249361
C	0.973609343355660	0.438135910131523	0.428141344025783
C	0.990534555040271	0.386859116694618	0.501955613813184
C	0.973604369672218	0.404605308241344	0.596231572441708
C	0.938452356062569	0.473740736950513	0.616863722901834
C	0.922244712273682	0.525229072844453	0.543554683501933
C	0.940066287866913	0.507775859453525	0.448888809553935
C	0.912644860022746	0.561810046762370	0.368775372250599
C	0.130066514351179	0.636993204026707	0.468770654931192
C	0.942125479296658	0.681697489751771	0.314032071430375
C	0.056995725875313	0.691465670913343	0.247887282444120
C	0.907811073971587	0.667368281864925	0.072684899129589
C	0.096303686665288	0.580002738385987	0.171253638610720
C	0.077036998601286	0.533215093461409	0.083567191264901
C	0.023349525036326	0.464123966421301	0.088838174744305
C	0.007051057206225	0.418977570757700	0.007788250857821
C	0.047982028454608	0.442095152079232	0.922273241082055
C	0.104057610089148	0.510486668043476	0.917384541356050
C	0.116696813372629	0.556433744263399	0.997254739391130
N	0.449481655223472	0.360767707640168	0.383748969884936
N	0.510597020722669	0.360539085123053	0.158991668005037
N	0.987288729858070	0.624079630190474	0.386373481873305
N	0.026380756209719	0.642934586150611	0.162554668915161
O	0.310415206741198	0.446278549590958	0.284929485267131
O	0.656246806579492	0.442530473793880	0.260760161857613
O	0.815377694923053	0.549848022688935	0.283195015245452
O	0.181381083904337	0.560950765092806	0.258560979470226
Cl	0.304883827470245	0.861407137706538	0.259418757114943
Cl	0.504974316688505	0.692477339584266	0.305997946914619

Cl 0.306933536432400 0.750879695622384 0.054576682180401
Cl 0.682977078882488 0.832059731175905 0.199493076077273
Cl 0.176828220002036 0.160427401760359 0.191007870155483
Cl 0.154296992305553 0.224463749412026 0.428827496714713
Cl 0.800298905524768 0.144020097840045 0.257887509816381
Cl 0.937355627301852 0.314136481123151 0.207081383611574
Fe 0.452093693604134 0.784403782559496 0.205116757651358
Fe 0.014705392802500 0.209599253392155 0.269976812527532

LBFGS Final Enthalpy = -2.86694141E+004 eV

[HL][$GaCl_4$]

Space group name: P2_1/c
Symmetry Int. Tables number: 14

Unit cell parameters (not optimised):
a = 8.673200000000000
b = 18.839199999999998
c = 13.864099570211474
α = 90.000000000000000
β = 104.665000552632065
γ = 90.000000000000000

Optimised symmetry-inequivalent fractional atomic coordinates:
H 0.000000000000000 0.000000000000000 0.000000000000000
H 0.500000000000000 0.000000000000000 0.000000000000000
H 0.834726392545944 0.190115643857110 0.244814259501562
H 0.972449386667875 0.165465760342477 0.173814683393024
H 0.904366624763531 0.101074476998058 0.246558786448959
H 0.677620433908988 0.866596427240918 0.736946282670482
H 0.625681900860548 0.091533371716543 0.283196824288513
H 0.819549556298452 0.245630917042253 0.943397228555718
H 0.944937212174227 0.183008204586261 0.020209036734505
H 0.724203460333276 0.946880783904108 0.883131083529159
H 0.716201332584932 0.878098533792710 0.391826882886294
H 0.934123244659224 0.101082119120055 0.724128017662833
H 0.646526706438542 0.003858381685814 0.418505698213420
H 0.734747436949292 0.208208887180502 0.762429316527198
H 0.576801280566105 0.191420670495468 0.820839971704140
H 0.636985072610978 0.124150615122875 0.748320782128066
H 0.739971775584128 0.928242002137502 0.093751448435743
H 0.761965310913457 0.841180375071804 0.229256249795621
H 0.761189145826356 0.902547955538459 0.585951884015557
H 0.700826304290738 0.237608562589001 0.076745647833568
H 0.581262222074236 0.178331488111642 0.990874933675901
H 0.894660250056854 0.019480673818773 0.580926840284373
C 0.869323375341128 0.147882858289598 0.200032995429436
C 0.735468364917635 0.917695335530320 0.734814048436052
C 0.856080665285103 0.077379635724999 0.900681480662159
C 0.657897971165155 0.036855737913035 0.272096128006748

C 0.684541522192930 0.016240776034872 0.180388374480803
C 0.823531646275104 0.191969026321946 0.974642639369794
C 0.761219919954192 0.962817647109435 0.816881398537187
C 0.665984850604079 0.068300173093167 0.098159412016998
C 0.706806178657411 0.917002173475724 0.332552443187876
C 0.879060464836657 0.049546167556663 0.727598193002225
C 0.668716043955745 0.987309060308269 0.347910219080945
C 0.679461239952237 0.167730346784438 0.799924011537870
C 0.719839684667983 0.945191951564958 0.164743084239506
C 0.731295016319567 0.896001376392524 0.241077238226496
C 0.782742402296975 0.938023071091769 0.649921236471031
C 0.831480536748374 0.029397912173502 0.813125192693712
C 0.701973606115434 0.187047166592633 0.037436472433296
C 0.856508561767795 0.003469157482470 0.646765343320311
N 0.737956694321501 0.131421085860697 0.113826524319881
N 0.792704246836832 0.142395716124154 0.890257993559168
O 0.936581982390851 0.057680127670164 0.987313016511179
O 0.578640912987813 0.053903196634624 0.010972237446571
Cl 0.080159623512720 0.264959795288051 0.815555081311277
Cl 0.442669293298347 0.347058898630196 0.968297650422062
Cl 0.065317247693771 0.360550132183231 0.032974061729808
Cl 0.277912766407973 0.193560277454704 0.056239743984643
Ga 0.219128125958009 0.292075015917457 0.969091822937917

LBFGS Final Enthalpy = -3.38179201E+004 eV

$[HL]_2[SnCl_6](H_2O)_2$

Space group name: P-1
Symmetry Int. Tables number: 2

Unit cell parameters (not optimised):
a = 8.772000000000000
b = 11.175200408318995
c = 12.075100537578848
α = 113.632000491744009
β = 103.282000928626815
γ = 97.176998704156475

Optimised symmetry-inequivalent fractional atomic coordinates:
H 0.500000000000000 0.500000000000000 0.500000000000000
H 0.000000000000000 0.500000000000000 0.500000000000000
H 0.216559120918237 0.441982043316907 0.337784760391829
H 0.151992019611722 0.368296169051380 0.107168611082445
H 0.265712841320790 0.519579665221669 0.025805643970445
H 0.449454306052828 0.745421038667423 0.178075893022785
H 0.512960012063610 0.819849705837839 0.410384258511932
H 0.378861720100692 0.966636637519482 0.581744653537135
H 0.215015146141580 0.901815216031839 0.623555942992183
H 0.225416409426162 0.819749050474370 0.466853954718685
H 0.489198287673090 0.947879996258088 0.788753385817458

H	0.556659268933833	0.800008615556469	0.766413749827218
H	0.359564941609794	0.861516327333972	0.900708507991048
H	0.205024233859345	0.814910153019159	0.758599145674163
H	0.530776193844094	0.686240714482467	0.886739431075334
H	0.401486362878104	0.527716994135017	0.841163890815748
H	0.382545671512864	0.678036960548894	0.963783046719759
H	0.205076461515977	0.325994328183834	0.498571323817395
H	0.158734226138033	0.101780541743700	0.496285763048065
H	0.068759020875645	0.072331995729653	0.668090258800592
H	0.023880145583241	0.266631755791986	0.838956035930306
H	0.074999180004460	0.492671203904751	0.842731242179220
H	0.266517672392577	0.908465489963218	0.286980401307284
H	0.247334375650146	0.049751324863363	0.315194286086035
C	0.266854753474293	0.509076746407635	0.303145611781948
C	0.230068145547064	0.467693039458442	0.172925279738740
C	0.294091970369116	0.553060013963659	0.127823806055913
C	0.396367711746813	0.679506846659686	0.212917152592841
C	0.432574731850394	0.721850810614631	0.343321861848913
C	0.366962518793253	0.636594792296549	0.388669240980103
C	0.411442058926285	0.676618700146629	0.527013526741098
C	0.297468560414572	0.874863279882048	0.566613161307864
C	0.449825298935599	0.837519288910542	0.744271155592460
C	0.321176957108718	0.797814877022447	0.797891597250332
C	0.408135338829124	0.634845381728569	0.872868027737217
C	0.180829808155058	0.560280567488775	0.678419653530901
C	0.145530338502308	0.423711003590255	0.672063588694390
C	0.165592189152234	0.313006919168000	0.573119529790262
C	0.138584155026359	0.186717987936624	0.571943833900058
C	0.089356162580614	0.170492125713828	0.668071303645681
C	0.064598294043010	0.280107524964153	0.765027985802895
C	0.093468181944585	0.406474258833716	0.767380056522118
N	0.390365024376673	0.791808299940074	0.606607713380993
N	0.294712856089167	0.657804056846855	0.778821496688914
O	0.471832163164137	0.600390279981743	0.571647364088815
O	0.104483774569878	0.587215333216554	0.590703485720287
O	0.301006372487177	0.999294762851927	0.355931788257565
Cl	0.106994195639769	0.161254146341044	0.230536669821643
Cl	0.144166654666459	0.834321271142015	0.037549998221538
Cl	0.766718487402660	0.897316407122071	0.040096407801890
Sn	0.000000000000000	0.000000000000000	0.000000000000000

LBFGS Final Enthalpy = -1.47667266E+004 eV

$[HL]_2[PtCl_6](H_2O)_2$

Space group name: P-1
Symmetry Int. Tables number: 2

Unit cell parameters (not optimised):
a = 8.757899999999999
b = 11.095399855137893

c = 12.066800022957329
α = 113.978001893642627
β = 103.941000153327565
γ = 96.912997591110440

Optimised symmetry-inequivalent fractional atomic coordinates:

H	0.500000000000000	0.500000000000000	0.500000000000000
H	0.000000000000000	0.500000000000000	0.500000000000000
H	0.217811414587838	0.438138583608378	0.334946058864228
H	0.154984774006060	0.364240723002482	0.103678354525033
H	0.266481806308654	0.519331416135698	0.025405568557410
H	0.446446375104950	0.749420374063027	0.181115012970291
H	0.510293056951501	0.824003467936949	0.414347362878144
H	0.218870880518270	0.908478698656836	0.627519697155964
H	0.225262669802989	0.822751820345774	0.469177857665693
H	0.381473561832280	0.971068953177174	0.583772697052141
H	0.496000940795044	0.953378946418060	0.793695311216642
H	0.559173481691915	0.802141827282571	0.770869977301719
H	0.361167062473632	0.870005657782743	0.904527208219937
H	0.207261865970220	0.821173166181995	0.759395308987466
H	0.532889591990130	0.693736286229190	0.894649055260574
H	0.402585577125366	0.533526228463607	0.847732818058322
H	0.382684795833305	0.685831092943428	0.969623568504202
H	0.074089122518076	0.497850217437452	0.843518600009403
H	0.025523578876555	0.271031295388618	0.842007418126682
H	0.070129172481487	0.074033917051352	0.672132760180784
H	0.157780068810326	0.099851955354017	0.498475774345195
H	0.206589346489288	0.324770615546537	0.500165321636966
H	0.735255781150493	0.089546766694144	0.714469992199268
H	0.755947040787150	0.947567907414000	0.685195603713698
C	0.267399639706231	0.507460373809254	0.302155270725125
C	0.231213004358047	0.465694975248159	0.171294636528995
C	0.294086001545451	0.553113268847941	0.127758518322052
C	0.394467862034065	0.681862283689248	0.214805272820672
C	0.430347222358187	0.724439251773737	0.345789197406449
C	0.366015875697237	0.637268917082033	0.389765273532289
C	0.411503525062508	0.678031031253851	0.528875533588958
C	0.299600639336751	0.879308900118835	0.569499223703089
C	0.453552125099728	0.841937582493970	0.748352260083328
C	0.323488811881921	0.804280449455852	0.801079416038641
C	0.409282276712772	0.641598427189817	0.878989551541461
C	0.181818516520876	0.563814416259167	0.680348173217583
C	0.146070819900577	0.426200970380516	0.673571106172118
C	0.093448862855331	0.410291147573719	0.768862423820659
C	0.065084141527083	0.283568232647716	0.767479176839104
C	0.089876681289799	0.172127851799099	0.670914596751495
C	0.138414682799324	0.186564306213935	0.574133000190287
C	0.166118953645678	0.313443136795035	0.574837013034244
N	0.392469501805197	0.795132904270445	0.609508695090289
N	0.295972057565497	0.663159105165649	0.782437385038920
O	0.471144467252231	0.600421585660461	0.573143489512787

O	0.105312100112303	0.589790906997898	0.591144647735132
O	0.703819455321788	-0.001415835618348	0.643408131369137
Cl	0.900449903947931	0.845725339016270	0.780460909736940
Cl	0.220866237168104	0.103245760866008	0.963159243730641
Cl	0.140180119877707	0.843997906706473	0.037249991842775
Pt	0.000000000000000	0.000000000000000	0.000000000000000

LBFGS Final Enthalpy = -2.57547264E+004 eV

[H_2O] (ice I_h)

Space group name: P 63 c m
Symmetry Int. Tables number: 185

Unit cell parameters (not optimised):
a = 7.820000000000000
b = 7.820000296532028
c = 7.360000000000001
α = 90.000000000000000
β = 90.000000000000000
γ = 119.999998745626570

Optimised symmetry-inequivalent fractional atomic coordinates:

H	0.330659901080247	0.330659906787303	0.691874401856240
H	0.448377776156737	0.448377796681465	0.515947716415078
H	0.782466648395504	0.665233389103451	0.477603357106534
O	0.326635759171375	0.326635764878430	0.556298784463969
O	0.660411624958412	0.660411619251357	0.430672383051645

LBFGS Final Enthalpy = -5.67215775E+003 eV

[Na][$AuCl_4$]

Space group name: P1
Symmetry Int. Tables number: 1

Unit cell parameters (not optimised):
a = 40.414518999999999
b = 40.414518999999999
c = 40.414518999999999
α = 90.000000000000000
β = 90.000000000000000
γ = 90.000000000000000

Optimised symmetry-inequivalent fractional atomic coordinates:

Na	0.000000000000000	0.000000000000000	0.000000000000000
Cl	0.464426818800000	0.525936891700000	0.464791922800000
Cl	0.535573181100000	0.474063108300000	0.535208077200000
Cl	0.542675702900000	0.529137944300000	0.477450640900000
Cl	0.457324297100000	0.470862055700000	0.522549359100000
Au	0.500000000000000	0.500000000000000	0.500000012400000

Dispersion Corrected Final Energy = -17126.95354197 eV

[Na][$FeCl_4$]

Space group name: P1
Symmetry Int. Tables number: 1

Unit cell parameters (not optimised):
a = 46.188022000000000
b = 46.188022000000000
c = 46.188022000000000
α = 90.000000000000000
β = 90.000000000000000
γ = 90.000000000000000

Optimised symmetry-inequivalent fractional atomic coordinates:
Na 0.000000 0.000000 0.000000
Cl 0.537778 0.534730 0.477156
Cl 0.504107 0.465270 0.463621
Cl 0.520665 0.489287 0.536379
Cl 0.462222 0.522729 0.494255
Fe 0.505801 0.503107 0.492771

Dispersion Corrected Final Energy = -3863.123470589 eV

[Na][$GaCl_4$]

Space group name: P1
Symmetry Int. Tables number: 1

Unit cell parameters (not optimised):
a = 46.188022000000000
b = 46.188022000000000
c = 46.188022000000000
α = 90.000000000000000
β = 90.000000000000000
γ = 90.000000000000000

Optimised symmetry-inequivalent fractional atomic coordinates:
Na 0.000000 0.000000 0.000000
Cl 0.519300 0.494302 0.463890
Cl 0.461872 0.524374 0.507974
Cl 0.538128 0.534597 0.525504
Cl 0.503146 0.465403 0.536110
Ga 0.505271 0.504752 0.508498
Dispersion Corrected Final Energy = -5149.896706651 eV

[Na]$_2$[SnCl$_6$]

Space group name: P1
Symmetry Int. Tables number: 1

Unit cell parameters (not optimised):
a = 46.188022000000000
b = 46.188022000000000
c = 46.188022000000000
α = 90.000000000000000
β = 90.000000000000000
γ = 90.000000000000000

Optimised symmetry-inequivalent fractional atomic coordinates:

Na	0.500000	0.000000	0.000000
Na	0.000000	0.000000	0.500000
Cl	0.487811	0.496767	0.447426
Cl	0.512189	0.503233	0.552574
Cl	0.547507	0.475644	0.491509
Cl	0.452493	0.524356	0.508491
Cl	0.523578	0.546948	0.490811
Cl	0.476422	0.453052	0.509189
Sn	0.500000	0.500000	0.500000

Dispersion Corrected Final Energy = -7213.363312638 eV

[Na]$_2$[PtCl$_6$]

Space group name: P1
Symmetry Int. Tables number: 1

Unit cell parameters (not optimised):
a = 46.188022000000000
b = 46.188022000000000
c = 46.188022000000000
α = 90.000000000000000
β = 90.000000000000000
γ = 90.000000000000000

Optimised symmetry-inequivalent fractional atomic coordinates:

Na	0.500000	0.000000	0.000000
Na	0.000000	0.000000	0.500000
Cl	0.511590	0.501931	0.549634
Cl	0.488410	0.498069	0.450366
Cl	0.476331	0.455616	0.508329
Cl	0.523669	0.544384	0.491671
Cl	0.544596	0.476455	0.491417
Cl	0.455404	0.523545	0.508583
Pt	0.500000	0.500000	0.500000

Dispersion Corrected Final Energy = -18201.53010929 eV

Section 6: Python script for preparing model unit cells

```
# ################################################################################
### IMPORT PACKAGES ##########################################################
print ('--- importing packages ---')
print ()
# Atomic Sumilation Environment
import ase
from ase import Atoms
from ase.io import read , write
# Mathematics
import numpy as np
from numpy import arccos , radians
import skspatial
from skspatial.objects import Line , Point
# Object manipulation
import copy
# User interface
import sys
import warnings # suppress CASTEP warnings when CASTEP not installed
from tkinter import *
from tkinter import filedialog # import files
from pyfiglet import figlet_format # print script name
from termcolor import cprint , colored # coloured output in terminal
from colorama import init , Fore , Style
init(convert=True , strip=not sys.stdout.isatty ()) # initialises colouring
# ################################################################################
### DISPLAY SCRIPT NAME ######################################################
cprint(figlet_format(' SIMULATION BOX BUILDER ', font='slant '), 'cyan')
# ################################################################################
### FILE BROWSER FOR USER TO SELECT A FILE ###################################
cprint('PICK A .CIF (or another ASE -supported file)', 'red')
input('A pop -up window will open where you can search for your file. '
'Click Enter to continue.')
print(Style.RESET_ALL)
# use tkinter to create a file browser window
file_not_selected = True
while file_not_selected:
root = Tk()
root.title('File Picker ')
root.geometry('500 x10') # root window , small because not necessary
filename = filedialog.askopenfilename(title="File Picker")
root.destroy ()
# check if a file was selected; file selection is not forced
if len(filename) != 0:
file_not_selected = False
else:
cprint('File not selected. Try again.', 'red')
print ()

# print the path to the file
print(colored('File chosen: ', 'cyan'), colored(filename , 'yellow '))
print(Style.RESET_ALL)
# ################################################################################
### ASK ABOUT THE FRAGMENT TO ISOLATE ########################################
cprint('WHAT IS THE DIMENSIONALITY OF THE FRAGMENT TO ISOLATE?', 'red')
```

```
print('Input 0 (e.g. metalate) or 1 (e.g. coil).')
print ()
dim_not_given = True
while dim_not_given:
cprint('Dimensionality = ', 'cyan ', end='')
dimension_input = input(Fore.YELLOW)
dimensionality = int(dimension_input) # interpret user input
dim_not_given = False
print(Style.RESET_ALL)
# ###########################################################################
### ASK FOR DISTANCE TO SET ##############################################
cprint('SET COUNTERION SEPARATION ', 'red')
print('Set the distance between the fragment and the counterions.')
print ()
dist_not_given = True
while dist_not_given:
cprint('Distance = ', 'cyan ', end='')
distance_input = input(Fore.YELLOW)
dist = float(distance_input) # interpret user input
dist_not_given = False
print(Style.RESET_ALL)
# ###########################################################################
### IF COIL , ASK FOR ORIENTATION ########################################
if dimensionality == 1:
cprint('GIVE COIL ORIENTATION ', 'red')
print('Set the crystallographic axis aligned with the direction of the coil (a/b/c).')
print ()
abc_dict = {'a':0, 'b':1, 'c':2} # interprets coil axis
pq_dict = {0:[1 ,2] , 1:[0,2], 2:[0 ,1]} # keep other axes as variables
coil_axis_not_selected = True
while coil_axis_not_selected:
cprint('Coil axis = ', 'cyan ', end='')
coil_axis_input = input(Fore.YELLOW)
# interpret user input
if coil_axis_input in abc_dict.keys():
# define coil , p and q axes
coil_axis = abc_dict[coil_axis_input]
p_axis = pq_dict[coil_axis ][0]
q_axis = pq_dict[coil_axis ][1]
coil_axis_not_selected = False
else:
cprint('Incorrect coil axis! Try again.', 'red')
print ()
print(Style.RESET_ALL)
# ###########################################################################
### READ THE FILE AND CREATE VARIABLES ###################################
with warnings.catch_warnings ():
warnings.simplefilter('ignore ')
atoms = ase.io.read(filename) # read structural information into ASE
symbols = atoms.get_chemical_symbols () # chemical symbols list
cell_lengths = atoms.get_cell ().lengths () # unit cell lengths list
cell_angles = [90, 90, 90] # unit cell angles set to 90 degrees
# if coil , set the unconstrained axes to desired distance
if dimensionality == 1:
cell_lengths[p_axis] = dist
```

```
cell_lengths[q_axis] = dist
# if metalate , set all cell axes to desired distance
if dimensionality == 0:
L = (2 * dist)/np.sqrt (3)
cell_lengths = [L, L, L]
# pass new cell parameters to the Atoms object
new_cell_params = np.concatenate (( cell_lengths , cell_angles))
atoms.set_cell(new_cell_params)
atoms.center ()
atoms.edit()
# get parameters to write to the .cell file
cell_matrix = atoms.get_cell () # unit cell vectors
cart_positions = atoms.get_positions () # cartesian positions list
frac_positions = atoms.get_scaled_positions () # fractional positions list
# ##############################################################################
### IF COIL , GIVE INFORMATION ABOUT COIL WIDTH ###########################
if dimensionality == 1:
frac_positions_to_fit_list = list ()
cart_positions_to_check = list ()
for i in range(0, len(symbols)):
if symbols[i] in {'C', 'N', 'O', 'H'}:
frac_positions_to_fit_list.append(frac_positions[i])
cart_positions_to_check.append(cart_positions[i])
# linear fit through C, N and O atom coordinates in the coil
frac_positions_to_fit = np.array(frac_positions_to_fit_list)
avg_coil_frac = np.mean(frac_positions_to_fit , axis =0)
avg_coil_cart = cell_matrix.cartesian_positions(avg_coil_frac)
coil_line = Line(point=Point(avg_coil_cart), direction=cell_matrix[
coil_axis ])
# find the furthest atom from the central axis of the coil
coil_radius = 0
for i in range(0, len(cart_positions_to_check)):
distance = coil_line.distance_point(cart_positions_to_check[i])
if distance > coil_radius:
coil_radius = distance
cprint('At its widest , the coil has a radius of: ', 'cyan', end='')
cprint("{:.6f}".format(coil_radius), 'yellow ', end='')
cprint(' angstroms.', 'cyan ')
print(Style.RESET_ALL)
# ##############################################################################
### WRITE OUTPUT .CELL FILE ################################################
if dimensionality ==1: # add counterions for coil
# set counterion 1
counter_ion_1 = np.zeros (3) # in the pc plane
counter_ion_1[q_axis] = 0.00
counter_ion_1[p_axis] = 0.50
counter_ion_1[coil_axis] = 0.50
# set counterion 2
counter_ion_2 = np.zeros (3) # in the qc plane
counter_ion_2[q_axis] = 0.50
counter_ion_2[p_axis] = 0.00
counter_ion_2[coil_axis] = 0.50
# write counterion positions and symbols
frac_positions_to_write = [counter_ion_1 , counter_ion_2]
symbols_to_write = ['Cl', 'Cl']
# set output file name
```

```
new_filename = f'{filename.split (".") [0]} _out_{dist}.cell'
elif dimensionality == 0: # add counterions for metalate
counter_ion = [0,0,0] # set counterion
# write counterion position and symbol
frac_positions_to_write = [counter_ion]
symbols_to_write = ['Na']
# set output file name
new_filename = f'{filename.split (".") [0]} _out_{dist}.cell'
for position in atoms.get_scaled_positions ():
frac_positions_to_write.append(position)
for symbol in atoms.get_chemical_symbols ():
symbols_to_write.append(symbol)
# write new cell file
with open(new_filename , 'w') as cell_file:
# write cell vectors
cell_file.write('%BLOCK lattice_cart ')
cell_file.write('\n')
cell_file.write("{:.6f}".format(cell_matrix [0 ,0]) + '\t'
+ "{:.6f}".format(cell_matrix [0 ,1]) + '\t'
+ "{:.6f}".format(cell_matrix [0 ,2]))
cell_file.write('\n')
cell_file.write("{:.6f}".format(cell_matrix [1 ,0]) + '\t'
+ "{:.6f}".format(cell_matrix [1 ,1]) + '\t'
+ "{:.6f}".format(cell_matrix [1 ,2]))
cell_file.write('\n')
cell_file.write("{:.6f}".format(cell_matrix [2 ,0]) +'\t'
+ "{:.6f}".format(cell_matrix [2 ,1]) + '\t'
+ "{:.6f}".format(cell_matrix [2 ,2]))
cell_file.write('\n')
cell_file.write('%ENDBLOCK lattice_cart ')
cell_file.write('\n')
cell_file.write('\n')
# write fix all cell boolean
cell_file.write('FIX_ALL_CELL : TRUE ')
cell_file.write('\n')
cell_file.write('\n')
# write atomic coordinates
cell_file.write('%BLOCK positions_frac ')
cell_file.write('\n')
for i in range(0, len(frac_positions_to_write)):
cell_file.write(symbols_to_write[i] + '\t' +
"{:.10f}".format(frac_positions_to_write[i][0]) + '\t' +
"{:.10f}".format(frac_positions_to_write[i][1]) + '\t' +
"{:.10f}".format(frac_positions_to_write[i][2]))
cell_file.write('\n')
cell_file.write('%ENDBLOCK positions_frac ')
cell_file.write('\n')
cell_file.write('\n')
# write species pot
cell_file.write('%BLOCK species_pot ')
cell_file.write('\n')
cell_file.write('%ENDBLOCK species_pot ')
cell_file.write('\n')
cell_file.write('\n')
# write symmetry generate command
cell_file.write('SYMMETRY_GENERATE ')
```

```
cell_file.write('\n')
cell_file.write('\n')
# write k-point mp spacing
cell_file.write('KPOINT_MP_SPACING : 0.05 ')

print(colored('The output file has been written to: ', 'cyan '),
colored(filename , 'green '))
print(Style.RESET_ALL)
```

Section 7: Crystallographic Tables

$[H\mathbf{L}]_2[SnCl_6]$

Chemical formula	$Cl_6Sn{\cdot}C_{36}H_{42}N_4O_4$
M_r	926.12
Crystal system, space group	Triclinic, $P\bar{1}$
Temperature (K)	120
a, *b*, *c* (Å)	8.5015 (1), 10.9005 (2), 11.7800 (2)
α, β, γ (°)	108.290 (2), 97.039 (1), 106.770 (2)
V ($Å^3$)	964.78 (3)
Z	1
Radiation type	Cu *K*α
μ (mm^{-1})	9.45
Crystal size (mm)	0.10 × 0.09 × 0.03 × 0.04 (radius)
Data collection	
Diffractometer	SuperNova, Dual, Cu at home/near, Atlas
Absorption correction	For a sphere<br>*CrysAlis PRO* 1.171.42.81a (Rigaku Oxford Diffraction, 2023) Spherical absorption correction using equivalent radius and absorption coefficient. Empirical absorption correction using spherical harmonics, implemented in SCALE3 ABSPACK scaling algorithm.
T_{min}, T_{max}	0.653, 0.675
No. of measured, independent and observed [$I > 2\sigma(I)$] reflections	28259, 4022, 4003
R_{int}	0.040
$(\sin \theta/\lambda)_{max}$ ($Å^{-1}$)	0.629
Refinement	
$R[F^2 > 2\sigma(F^2)]$, $wR(F^2)$, S	0.018, 0.047, 1.02
No. of reflections	4022
No. of parameters	236
H-atom treatment	H atoms treated by a mixture of independent and constrained refinement
$\Delta\rangle_{max}$, $\Delta\rangle_{min}$ (e $Å^{-3}$)	0.63, -0.49

[H**L**][$GaCl_4$]

Chemical formula	$Cl_4Ga{\cdot}C_{18}H_{20.5}N_2O_2$
M_r	508.38
Crystal system, space group	Monoclinic, $P2_1/n$
Temperature (K)	120
a, *b*, *c* (Å)	8.6732 (2), 18.8392 (7), 13.8641 (5)
β (°)	104.665 (3)
V ($Å^3$)	2191.55 (13)
Z	4
Radiation type	Mo *K*α
μ (mm^{-1})	1.76
Crystal size (mm)	0.56 × 0.14 × 0.11
Data collection	
Diffractometer	Xcalibur, Eos
Absorption correction	Multi-scan<br>*CrysAlis PRO* 1.171.41.99a (Rigaku Oxford Diffraction, 2021) Empirical absorption correction using spherical harmonics, implemented in SCALE3 ABSPACK scaling algorithm.
T_{min}, T_{max}	0.502, 1.000
No. of measured, independent and observed [$I > 2\sigma(I)$] reflections	45977, 5157, 4560
R_{int}	0.065
$(\sin \theta/\lambda)_{max}$ ($Å^{-1}$)	0.668
Refinement	
$R[F^2 > 2\sigma(F^2)]$, $wR(F^2)$, S	0.051, 0.121, 1.22
No. of reflections	5157
No. of parameters	250
H-atom treatment	H atoms treated by a mixture of independent and constrained refinement
$\Delta\rangle_{max}$, $\Delta\rangle_{min}$ (e $Å^{-3}$)	0.99, -0.78

References

1. Bauer, L., Reductive Lossen Rearrangement Induced by Lithium Aluminum Hydride. *J Am Chem Soc* **1956,** *78* (9), 1945-1946.
